# Design and synthesis of a ratiometric photoacoustic imaging probe activated by selenol for visual monitoring of pathological progression of autoimmune hepatitis†

**DOI:** 10.1039/d0sc06573k

**Published:** 2021-02-19

**Authors:** Chaobang Zhang, Zhidong Qiu, Liangliang Zhang, Qiufang Pang, Zhengmin Yang, Jiang-Ke Qin, Hong Liang, Shulin Zhao

**Affiliations:** State Key Laboratory for the Chemistry and Molecular Engineering of Medicinal Resources, School of Chemistry and Pharmaceutical Sciences, Guangxi Normal University Guilin 541004 China liangzhang319@163.com zhaoshulin001@163.com

## Abstract

Photoacoustic (PA) imaging with both the high contrast of optical imaging and the high spatial resolution of ultrasound imaging has been regarded as a robust biomedical imaging technique. Autoimmune hepatitis (AIH) is the second largest liver inflammatory disease after viral hepatitis, but its pathogenesis is not fully understood probably due to the lack of an effective *in vivo* monitoring approach. In this work, an innovative selenol-activated ratiometric PA imaging probe APSel was developed for visual monitoring of pathological progress of AIH. Selenols including selenocysteine (Sec, the major form of Se-containing species *in vivo*) have been demonstrated to have an effective antioxidant role in inflammation. The reaction of APSel with selenol results in a blue shift of the PA spectrum peak from 860 nm to 690 nm, which enables the ratiometric PA imaging. The APSel probe displays high sensitivity and selectivity to Sec and other selenols. The APSel probe was then employed for ratiometric PA imaging of selenol in cells, and for monitoring the development of AIH in a murine model by tracking the changes of selenol level. The results revealed that the level of selenol was closely correlated with the development of AIH. The proposed APSel, as the first example of a selenol-responsive PA imaging probe, provides a new tool and approach to study and diagnose AIH diseases.

## Abstract 2

A ratiometric photoacoustic imaging probe activated by selenol was developed for visual monitoring of pathological progression of autoimmune hepatitis.

## Graphical abstract

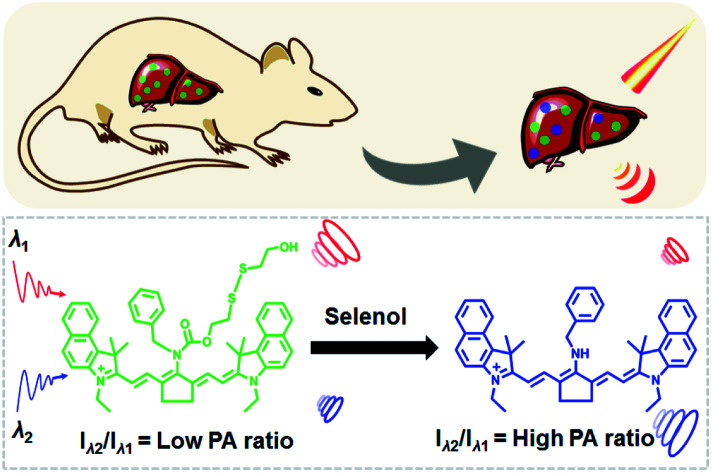

## Introduction

Selenium is an essential micronutrient for the human body and plays an important role in the prevention and treatment of cancer and cardiovascular diseases.^1^ Selenium exists in various chemical forms *in vivo*, such as selenocysteine (Sec), selenoglutathione (GSeH), selenomethionine and methylselenocysteine.^2,3^ These active selenium species together with the active sulfur species (cysteine, glutathione, and homocysteine) form part of the cellular antioxidant system, which can effectively remove the excess reactive oxygen species generated during the development of inflammation, prevent cellular oxidative damage, and maintain the cellular redox equilibrium.^4^ Among the active selenium species, Sec is the main form of selenium,^5^ which is a cysteine analogue with a selenium-containing selenol group. Sec is directly involved not only in antioxidant stress, but also in the synthesis of various selenium-containing antioxidants.^6^ Sec is also an important component of selenoproteins, which are involved in various cellular functions and are associated with some human diseases.^7^ Since Sec performs the majority of functions of Se-containing species,^5^ monitoring Sec *in vivo* is critical for understanding the physiological role and studying the mechanism of selenium species.

Autoimmune hepatitis (AIH), an idiopathic syndrome mediated by immune cells that destroy hepatocytes, is usually associated with the formation of autoantibodies and regulatory T cell dysfunction.^8,9^ AIH has become the second largest liver inflammatory disease after viral hepatitis. However, the pathogenesis of the AIH disease is not fully understood and there is a lack of effective and accurate diagnostic methods for this disease.^10^ A large amount of reactive oxygen species is produced during the development of inflammation. As a kind of major antioxidant molecule like biothiol, selenol may also be closely associated with the pathogenesis of AIH. However, due to the unstable chemical properties of selenol, the synthesis of selenol-specific probes is challenging. This has led to the lack of a probe for monitoring selenol *in vivo*, and limited the study of physiological and pathological mechanisms of selenol. Antibodies to soluble liver antigen (SLA) and liver pancreas (LP) antigen are important AIH-specific antibodies that have been used as diagnostic markers.^11^ It is noteworthy that SLA and LP are also the most important Sec synthases.^12–14^ Therefore, due to the relationship between Sec and AIH,^14^ monitoring selenol including Sec during the progress of AIH may provide a new approach to study the pathogenesis of the AIH disease and its diagnosis.

The photoacoustic (PA) imaging technique, which combines the advantages of optical imaging and acoustic imaging, has both the high contrast of optical imaging and the high spatial resolution of ultrasound imaging. It can overcome the soft limit of traditional optical imaging, achieve high-resolution imaging at the cm-level depth, and enable high-resolution visualization in deep tissue.^15,16^ Additionally, it also has the advantages of using non-ionizing radiation and being non-invasive, as well as having no effect on normal tissue.^17–19^ At present, the main method for AIH diagnosis is to detect biomarkers in serum.^20^ However, many biomarkers in serum come not only from the liver, but also from other organs or tissues of organisms,^21^ which makes the detection of biomarkers in serum lack specificity. The merits of PA imaging enable it to be a robust approach for AIH monitoring by directly imaging liver tissue. Using a selenol-responsive PA imaging probe to image liver tissue may provide an alternative new approach for AIH monitoring. However, to date, there have been no reports of PA probes for selenol imaging. Therefore, developing a PA imaging probe for selenol detection and visual monitoring of AIH will help to gain an insight into the pathogenesis of AIH and ensure the accurate diagnosis of AIH.

**Fig. 1:**
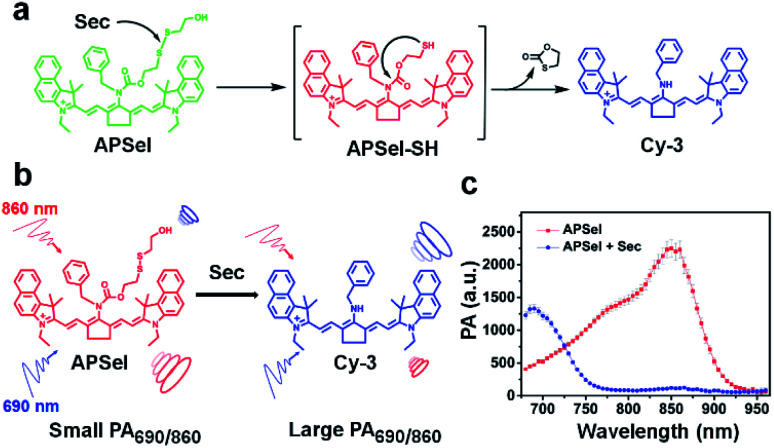
(a) Schematic diagram of the reaction of the APSel probe with Sec. (b) Principle of ratiometric PA detection of Sec. (c) PA spectra of APSel solution in the presence or absence of Sec.

In this work, we designed and synthesized the first selenol-responsive PA imaging probe (APSel) for detecting selenol levels *in vivo*, and we used it for the visual monitoring of the AIH disease progress. The probe is composed of a near-infrared cyanine dye and the selenol-responsive group bis(2-hydroxyethyl)disulfide. The selenium–sulfur exchange reaction of APSel and selenol (Sec was used here) causes the release of the responsive group in the APSel molecule, which results in a blue shift of the PA spectrum peak from 860 nm to 690 nm (Fig. 1). Thus, the PA signal at 860 nm weakens while the PA signal at 690 nm increases after the reaction. The ratio of the PA signal intensity at these two wavelengths (PA_690_/PA_860_) can then be used for quantifying selenol concentration, which enables the ratiometric PA imaging. Ratiometric imaging has a powerful self-correcting ability, which can eliminate the interference caused by the non-uniform accumulation and photobleaching of the probe in the target tissue *in vivo*.^22^ Therefore, accurate imaging of selenol *in vivo* can be achieved by using the prepared APSel as a ratiometric PA imaging probe, benefiting the effective monitoring of AIH.

## Results and discussion

### Design and synthesis of the PA probe for selenol

**Fig. 2:**
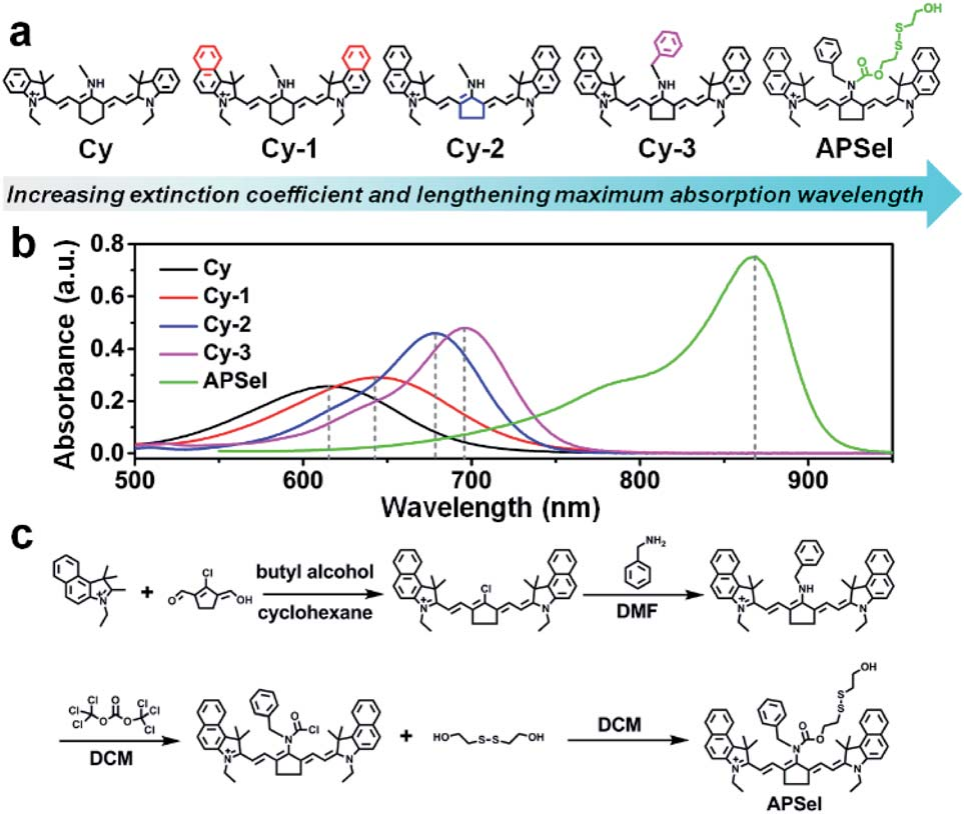
(a) The structural formulae of the probe precursors. (b) The absorption spectra of the APSel probe and its precursors. (c) The synthetic route to APSel.

In the human body, selenol has a high activity, occurs at low concentration, and has a structure similar to that of thiols. Such characteristics make the synthesis of specific selenol probes highly challenging.^23^ Selenol usually exists in its deprotonated form at physiological pH, and has strong nucleophilic properties.^6^ Exploiting such chemical properties of selenol, and considering that bis(2-hydroxyethyl)disulfide quickly responds to selenol with highly specificity to Sec and GSeH, bis(2-hydroxyethyl)disulfide was adopted as the responsive group of the PA probe for selenol.^24^ In addition, to minimize background interference from organisms and match the excitation bands of the PA imaging instruments, it is better that the maximum absorption wavelength of the PA probes is in the near infrared to infrared region.^25^ Accordingly, we employed the near-infrared cyanine dye as the scaffold to design the PA probe for selenol. We first prepared a series of probe precursors including Cy, Cy-1, Cy-2, and Cy-3 (structures were shown in Fig. 2a) to improve the absorption properties for constructing an ideal PA probe. The characterization of these probe precursors is shown in Fig. S1–S12 (ESI).† The probe precursor Cy has a maximum absorption wavelength (*λ*_max_) at 615 nm, as can be seen from Fig. 2b and Table S1 (ESI),† which is not an appropriate imaging wavelength for the *in vivo* PA detection. So, we then prepared other precursors Cy-1, Cy-2 and Cy-3 by the modification of Cy by introducing benzene rings, converting six-membered rings into five-membered rings, or converting methylamine into benzylamine, respectively (Fig. 2a). It was found that after strengthening the molecular conjugation system, the *λ*_max_ values of Cy-1 and Cy-2 were red shifted to 643 nm and 678 nm, respectively (Fig. 2b and Table S1, ESI†). And the yield of Cy-2 was increased compared to that of Cy-1, due to the fact that the coplanarity of the five-membered rings in the cyanine dye is better than that of the six-membered rings,^26^ and more stable than the pentacyclic carbon positive ions in the amine nucleophilic substitution reaction.^27^ Because of the lower electron density of benzylamine,^28^ compared to Cy-2, the *λ*_max_ for Cy-3 was further red shifted to 696 nm (Fig. 2b and Table S1, ESI†).

To achieve low *in vivo* background interference, we then selected Cy-3 as the precursor to construct the PA probe (APSel) for selenol by linking the selenol-responsive group bis(2-hydroxyethyl)disulfide. The synthetic route to APSel is shown in Fig. 2c. The characterization of the probe molecule including LC-MS, ^1^H NMR, and ^13^C NMR is shown in Fig. S13–S15 (ESI).† The *λ*_max_ of APSel shifted to 869 nm (Fig. 2b), and the absorption coefficients increased (Table S1, ESI†), which can provide a strong PA signal. The reaction mechanism of APSel to Sec is shown in Fig. 1a. The reduction of the disulfide bond in APSel by Sec initiated a rapid intramolecular cyclization, causing the cleavage of the adjacent amide bond and releasing the responsive group in the APSel molecule. Thereby, the resulting molecule is restored to Cy-3, and the PA spectrum peak is blue shifted (Fig. 1b). As we can see from the PA spectra in Fig. 1c, after adding Sec to APSel solution, the PA signal at 860 nm decreased while the signal at 690 nm increased. Thus, the ratiometric PA imaging can be achieved by recording the PA signals at 860 nm (from APSel) and 690 nm (from Cy-3, the product after the reaction of APSel with Sec), respectively. And these two signal peaks are located in the near infrared region; therefore, APSel with ratiometric imaging ability can be used for providing more accurate information about selenol *in vivo* due to the low background interference.

To verify the Sec-induced response mechanism, MS spectra of APSel and its reaction mixture after adding Sec were collected. The obtained MS results in Fig. S16 and S17† show a strong *m*/*z* peak at 668.40613 after APSel reacted with Sec, which was nearly identical to the molecular weight of Cy-3 (calcd [M]^+^: 668.39993). In addition, the *m*/*z* peak for the intermediate product (APSel-SH, shown in Fig. 1a) is also observed at 772.40033 (calcd [M]^+^: 772.39313). The above results verified the proposed response mechanism and demonstrated that APSel can be used as a probe for Sec.

### PA response performance of APSel to Sec in buffer

**Fig. 3:**
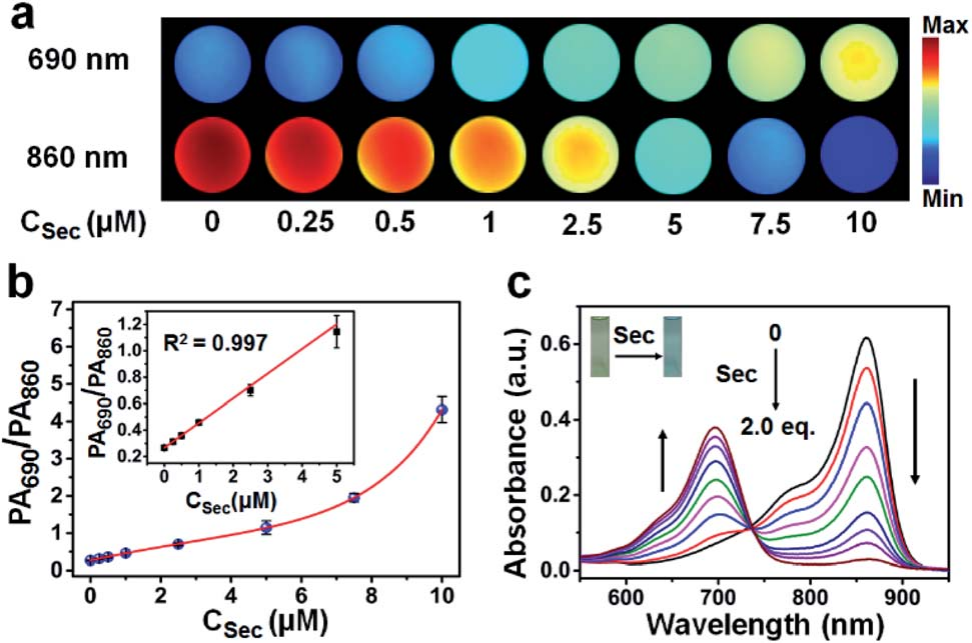
(a) PA imaging of the 5 μM APSel solutions at 690 nm and 860 nm in the presence of Sec at different concentrations. (b) The relationship between PA_690_/PA_860_ and Sec concentration. (c) Absorption spectra of 5 μM APSel solution after adding Sec at different concentrations. The inset is the photograph of the APSel solution before and after the reaction.

The response performance of APSel to selenol was first tested by recording the PA imaging signal after adding Sec to APSel-containing HEPES buffer solution. From the images shown in Fig. 3a, we can find that with the increase of Sec concentration, the PA imaging signal was increased at 690 nm and weakened at 860 nm gradually (Fig. 3a). The PA signal intensity ratio (PA_690_/PA_860_) was significantly increased with an increase of Sec concentration (Fig. 3b). It showed a linear relationship in the concentration range of 0–5 μM, and the detection limit was estimated to be 73 nM. Serum concentrations of selenium have been found to be higher than 0.5 μM in healthy adults,^23^ indicating that the APSel probe has the potential to detect Sec *in vivo*. The UV-vis absorption spectra (Fig. 3c) also demonstrated a blue shift of *λ*_max_ when adding Sec to APSel solution, accompanied by a decrease of absorbance at 860 nm and an increase of absorbance at 690 nm. At the same time, the solution color changed from green to blue (inset of Fig. 3c).

### Stability and selectivity of APSel to Sec

**Fig. 4:**
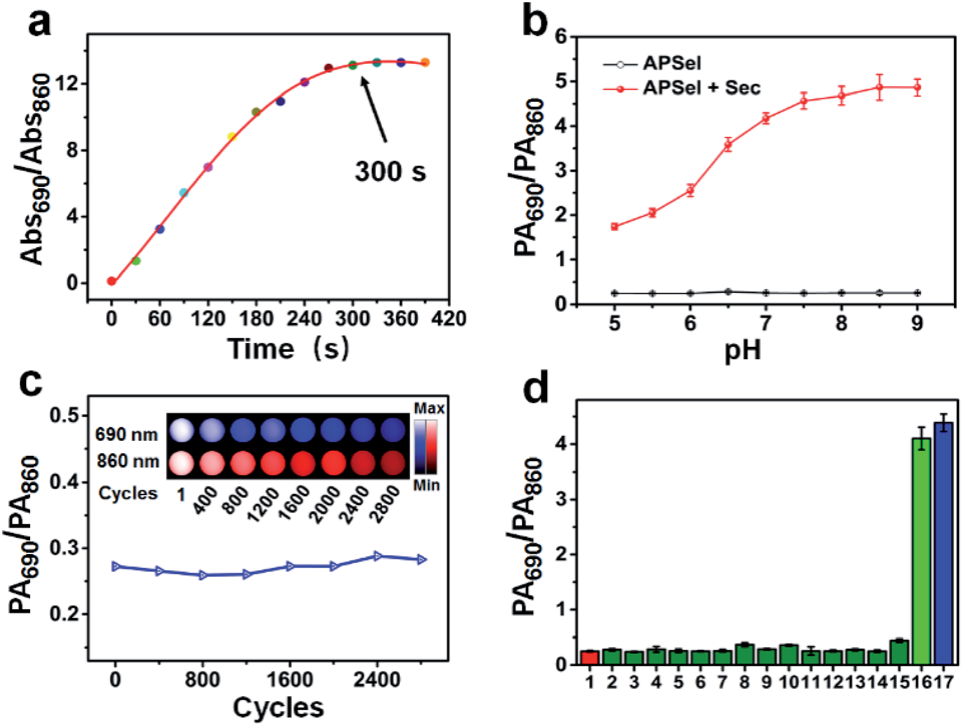
(a) Time-dependent response in absorbance ratio at 690 nm and 860 nm (Abs_690_/Abs_860_) after adding 10 μM Sec to 5 μM APSel solution. (b) The effect of pH on the PA_690_/PA_860_ value of APSel in the presence or absence of Sec. (c) The change of PA signal intensity and ratio for APSel solution at 690 nm and 860 nm under continuous cyclic scanning. (d) Selectivity tests of APSel. (1) Blank; (2) GSH (5 mM); (3–5) Cys, Hcy, Na_2_S (each 100 μM); (6–17) Cys-SSH, NAC, DTT, GPx, TrxR, Se-methylselenocysteine, selenocystine, selenomethionine, Na_2_Se, Na_2_SeO_3_, GSeH, Sec (each 10 μM).

The prepared APSel probe showed a fast response to Sec, and the reaction between APSel and Sec was complete within 300 s (Fig. 4a), implying that APSel can be used for real-time PA imaging of selenol *in vivo*. The stability of APSel and its reaction product with Sec was then investigated by recording their absorption spectra (Fig. S18, ESI†). The results revealed no obvious decomposition in either APSel or its reaction product within 12 h. Moreover, APSel also showed good stability in the pH range of 5–9. The PA_690_/PA_860_ value increased with the increase of the pH value after the reaction of APSel with Sec, as a result of the Sec deprotonation promoted by high pH, which is beneficial to the exchange of selenium and sulfur bonds. The photostability of APSel was also investigated by recording the PA signal under continuous cyclic scanning. The results revealed a decrease in PA signal intensity of APSel both at 690 nm and 860 nm probably due to the decomposition, but the PA_690_/PA_860_ value remained essentially unchanged (Fig. 4c). All these results indicate that APSel has sufficient stability for *in vivo* accurate PA imaging.

The selectivity of the APSel for selenol was evaluated by examining the response of other species including biothiols, Na_2_S, DTT, and active selenium species to the probe, and the results are shown in Fig. 4d. It reveals that among the bioactive compounds tested, only selenol (Sec and GSeH) can induce a significant increase in the PA_690_/PA_860_ ratio, while other species did not significantly respond to the probe. In addition, APSel was inert to active oxygen, reactive nitrogen, metal ions and sulfur-free amino acids (Fig. S19, ESI†). The above results demonstrated that APSel has good specificity for selenol, having potential for application in the complex *in vivo* biological system.

### Ratiometric PA imaging of endogenous selenol in cells

**Fig. 5:**
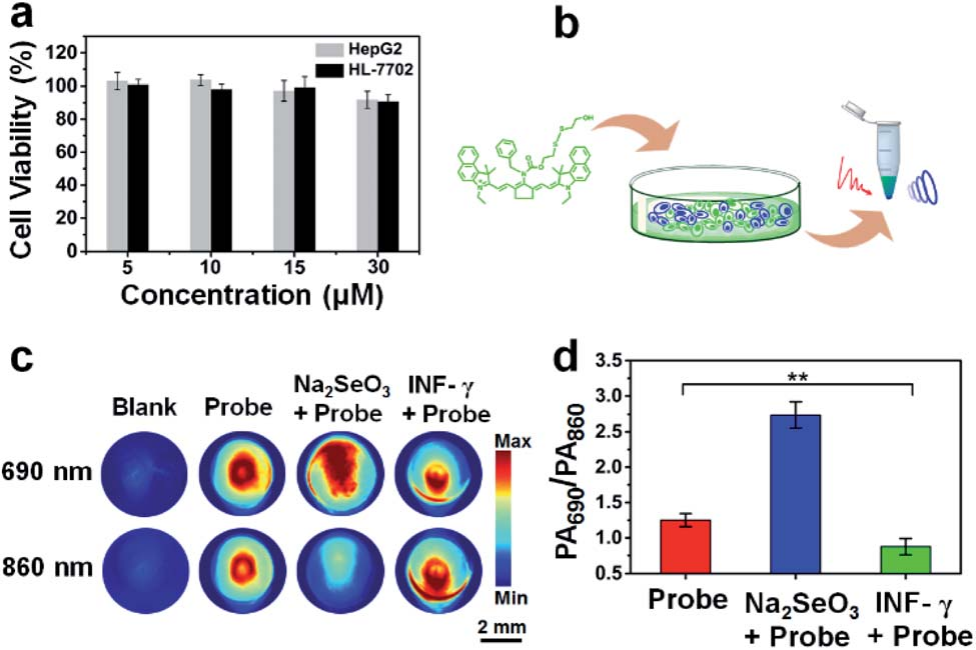
(a) The cell viability of HepG2 and HL-7702 cells after incubation with APSel at different concentrations. (b) The schematic illustration of cell pellet preparation. (c) Representative PA imaging of HL-7702 cell pellets under different conditions: blank; probe (5 μM APSel, incubated for 1 h); Na_2_SeO_3_ (5 μM, incubated for 24 h) + probe (5 μM APSel, incubated for 1 h); INF-γ (60 ng mL^−1^, incubated for 24 h) + probe (5 μM APSel, incubated for 1 h). (d) The PA_690_/PA_860_ values correspond to (c). The error bar represents the standard deviation, *n* = 3 independent cell pellets. The statistical analysis was performed using the Student's *t*-test method, **p < 0.01.

The cell viability test demonstrated that APSel had low cytotoxicity (Fig. 5a), which benefited the PA imaging of selenol in cells. For the PA imaging of selenol in cells, we pretreated HL-7702 cells with Na_2_SeO_3_, a precursor for biosynthesis of Sec,^29^ followed by incubation with APSel. Then the cells were centrifuged to obtain the cell pellets for PA imaging (Fig. 5b). The obtained PA images and PA_690_/PA_860_ values are displayed in Fig. 5c and d, respectively. We can find that when cells were pretreated with Na_2_SeO_3_, compared to sole incubation of the APSel probe, the PA signal of the cell pellets increased at 690 nm and decreased at 860 nm, and thus the PA_690_/PA_860_ value increased from 1.25 to 2.74. Since the PA signal background of the cell pellets is negligible, the increase of the PA_690_/PA_860_ value reflects the response result of APSel to high concentration of intracellular Sec. As Na_2_SeO_3_ is a precursor for biosynthesis of Sec, preincubation with Na_2_SeO_3_ enhanced the endogenous cellular selenol, resulting in the increase of the PA_690_/PA_860_ value, which demonstrates that the APSel probe can be used for PA imaging of endogenous cellular selenol. To further demonstrate this, INF-γ, the main proinflammatory cytokine,^30^ was employed for preincubation with HL-7702 cells followed by incubation with APSel. As illustrated in Fig. 5c and d, after INF-γ-induced inflammation, the PA_690_/PA_860_ value decreased to 0.87. This is attributed to the involvement of selenol in the anti-inflammatory response which leads to its consumption.^31^ These findings indicate that APSel has the potential to be used for ratiometric PA imaging of the changes of endogenous selenol levels.

### Ratiometric PA imaging of exogenous selenol *in vivo*

**Fig. 6:**
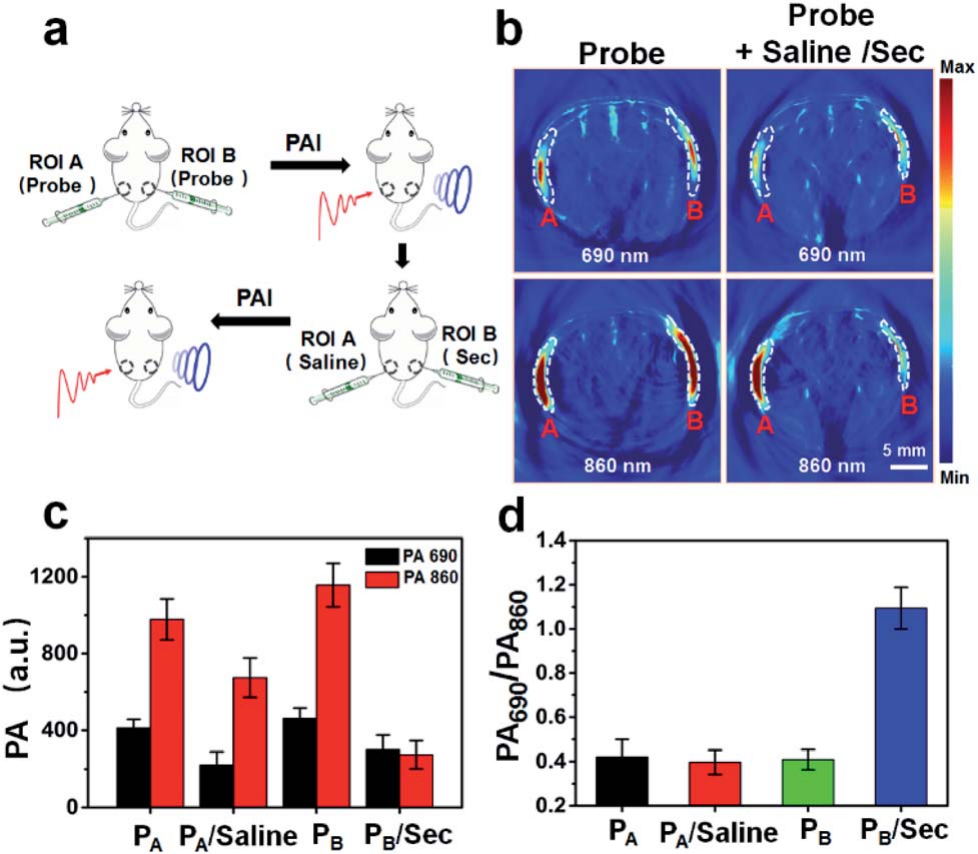
(a) Schematic illustration of *in vivo* PA imaging of exogenous selenol. PA imaging was performed after subcutaneous injection of APSel (15 μM, 100 μL) followed by injecting saline (100 μL) at ROI A, and Sec (30 μM, 100 μL) at ROI B. (b) Representative PA imaging of mice treated with different substances under excitation by light irradiation at 690 nm and 860 nm. (c) The PA signal values correspond to (b). (d) The PA_690_/PA_860_ values correspond to (b). The error bar represents the standard deviation, *n* = 3 independent mice.

The ability to achieve ratiometric PA imaging of selenol *in vivo* using APSel was assessed by subcutaneous injection of the probe and exogenous Sec (Fig. 6a). After injecting APSel, there was an obvious PA signal at 690 nm and 860 nm in the regions of interest (ROIs) A and B, and the PA signal is stronger at 860 nm (Fig. 6b and c). As expected, the PA_690_/PA_860_ values in ROIs A and B are close (Fig. 6d). Subsequently, we further injected saline and Sec into ROI A and ROI B, respectively. The PA signal at both wavelengths in ROI A and B was reduced. This is due to the reduction of the local concentration of the APSel probe by diffusion penetration and secondary injection dilution. However, the PA_690_/PA_860_ value in ROI B was increased from 0.41 to 1.09, while there was no significant change of the PA_690_/PA_860_ value in ROI A injected with saline (Fig. 6d). This confirms that the interference caused by the deviation of the local concentration of the probe can be overcome by ratiometric PA imaging, which achieved accurate detection of selenol *in vivo*.

### Monitoring the progress of AIH using the APSel probe

To perform PA imaging of endogenous selenol *in vivo* and monitor the disease process of AIH, the changes in the physiological status of mice after the tail vein injection of APSel were evaluated first. The results revealed that after injecting APSel, the mice did not die, suffer organ damage or experience weight loss, and only showed a slight increase in lymphocyte and neutrophil levels (Fig. S20–S22, ESI†). The probe accumulates mainly in the liver (Fig. S23, ESI†), which may be attributed to the hydrophobicity of the probe, thereby avoiding the rapid metabolism in the kidneys.^32^ However, strong background PA signal in the liver was observed probably from deoxyhemoglobin (Fig. S23–S25a, ESI†). Here, to reduce background interference, we used the PA signal increments (ΔPA, which is defined as the difference between the PA intensity of the treated group and that of the blank group) to obtain the PA signal ratio (ΔPA_690_/ΔPA_860_) for quantification.

**Fig. 7:**
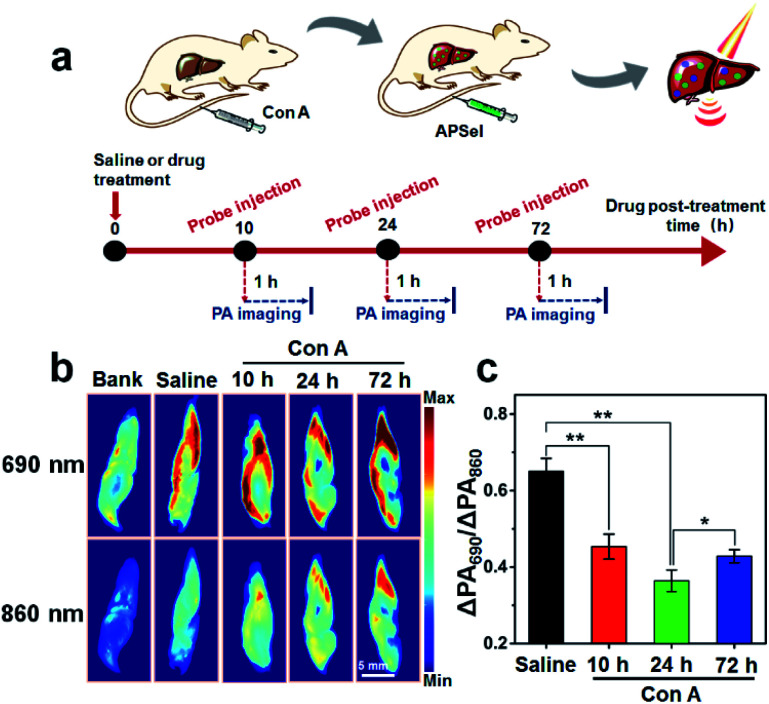
(a) Schematic illustration of monitoring the pathological progression of AIH using the PA probe. (b) Representative *ex vivo* PA images of isolated livers from mice under different conditions. Blank group: injected saline without any other agent; saline group: saline, 24 h, + APSel (0.1 mg kg^−1^), 1 h; Con A groups: Con A (15 mg kg^−1^), 10, 24, 72 h, + APSel (0.1 mg kg^−1^), 1 h. (c) The changes of the ΔPA_690_/ΔPA_860_ value for the liver in mice under different conditions. The error bar represents the standard deviation, *n* = 3 independent mice. The statistical analysis was performed using the Student's *t*-test method, *p < 0.05, **p < 0.01.

We established the AIH disease model by injecting concanavalin A (Con A) in mice here (Fig. 7a),^33^ and then adopted APSel to monitor the changes of selenol level in the liver during the development of AIH by ratiometric PA imaging (Fig. 7b). Although it is difficult to accurately assess the changes of selenol concentration by PA imaging of the mouse liver due to background PA signal, based on the quantitative analysis of ΔPA_690_/ΔPA_860_ in the Con A-induced AIH group compared to the normal saline-treated group (Fig. 7c), the ΔPA_690_/ΔPA_860_ value decreased from 0.65 to 0.45 at 10 h post-Con A injection, and further decreased to 0.36 at 24 h post-Con A. Besides the decrease of selenol content during inflammation through anti-inflammatory response, another major reason for the decrease may be the inhibition of Sec synthesis.^34^ The ΔPA spectra of the isolated liver further confirmed that the change of the ΔPA ratio was the result of the response of APSel to selenol (Fig. S25b, ESI†). The ΔPA_690_/ΔPA_860_ increased to 0.43 at 72 h post-Con A injection, and there was a statistically significant difference compared with the value at 24 h (Fig. 7c). This indicated an increase in selenol content, as the inflammation may be alleviated and mice may experience self-healing at low doses of Con A.^35^ These results indicate that as the AIH disease progresses, the content of selenol decreases significantly, and when inflammation was alleviated, the selenol content rebounds. The serum levels of the hepatitis markers AST and ALT were consistent with the PA imaging results, confirming the occurrence of inflammation (Fig. S26, ESI†). Histological analysis (Fig. S27a, ESI†) also revealed that inflammation occurred after injecting Con A within 24 h accompanied by the degeneration of hepatocytes and the increase of inflammatory cells, while at 72 h post-Con A injection, hepatocytes recovered and inflammatory cells decreased. Meanwhile, an increase in inflammation makes the liver dark (Fig. S27b, ESI†). There was no significant difference between the results of AIH disease pathogenesis analysis by AST/ALT detection, histological staining and liver entity imaging, which proved that the injection of APSel did not interfere with the development of Con A-induced AIH. These results further confirm that APSel can be used as an effective ratiometric PA imaging probe to monitor the development of the AIH disease.

## Conclusions

In conclusion, we have developed a ratiometric PA imaging probe APSel for selenol detection and monitoring the progress of AIH. APSel, which was prepared by using cyanine dye as the scaffold and using bis(2-hydroxyethyl)disulfide as the selenol-responsive group, has good stability, and fast, sensitive and selective response to selenol. In addition, the ratiometric PA imaging ability of APSel enables the accurate monitoring of selenol *in vivo*. This probe was employed for PA imaging of endogenous selenol in cells. And monitoring the development of the AIH disease in a Con A-induced AIH murine model by tracking the changes of selenol level was successfully achieved. To the best of our knowledge, APSel prepared in this work is the first example of a selenol-responsive PA probe, and a new method is provided for the diagnosis and pathogenesis studies of the AIH disease. Also, the design strategy for this probe also offers an effective way to develop PA probes in the near infrared biowindow.

## Ethical statement

All animal experiments were approved by the Animal Ethics Committee of Guangxi Normal University, and were conducted under the protocols of the Care and Use of Laboratory Animals of the Guangxi Normal University.

## Conflicts of interest

There are no conflicts to declare.

## Supplementary Material

SC-012-D0SC06573K-s001
