# Supplementary material for: Design and synthesis of a ratiometric photoacoustic imaging probe activated by selenol for visual monitoring of pathological progression of autoimmune hepatitis
Source: Chem Sci. 2021 Feb 19;12(13):4883–8. doi: 10.1039/d0sc06573k (PMC8179563; doi:10.1039/d0sc06573k)
Supplement: SC-012-D0SC06573K-s001 [file SC-012-D0SC06573K-s001.pdf]

Electronic Supplementary Material (ESI) for Chemical Science.
This journal is © The Royal Society of Chemistry 2021

# Electronic supplementary information (ESI)

## Design and synthesis of a ratiometric photoacoustic imaging probe activated by selenol for visual monitoring pathological progression of autoimmune hepatitis

Chaobang Zhang, Zhidong Qiu, Liangliang Zhang,* Qiufang Pang, Zhengmin Yang, Jiang-Ke Qin, Hong Liang, and Shulin Zhao*

State Key Laboratory for the Chemistry and Molecular Engineering of Medicinal Resources, School of Chemistry and Pharmaceutical Sciences, Guangxi Normal University, Guilin 541004, China

# EXPERIMENTAL SECTION

## Chemicals

All chemicals were purchased from commercial suppliers and used without further purification or modification. 2,3,3-trimethylindolenine, 1,1,2-trimethyl-1H-benz[e]indole, iodoethane, cyclopentanone, cyclohexanone, triethylamine, methylamine hydrochloride, phosphorus oxitrichloride, L-serine (Ser), α-lipoic acid (Ala), L-histidine (His), L-glutamic acid (Glu), benzylamine, boc-L-glutamic acid 1-tert-butyl ester, N,N-diisopropylethylamine, bis (2-hydroxyethyl) disulfide, hematoxylin, eosin, 4-dimethylaminopyridine, 3-morpholinosydnonimine hydrochloride, Se-methylselenocysteine, dimethyl sulfoxide (DMSO), selenocystine ($(CysSe)_2$), selenomethionine and N-acetyl-L-cysteine (NAC) were purchased from Beijing Bailingwei Technology Co. Ltd. (Beijing, China). Xanthine, xanthineoxidase, sodium selenide ($Na_2Se$), sodium selenite ($Na_2SeO_3$), agar, 1-hydroxy-2-oxo-3-(N-methyl-3-aminopropyl)-3-methyl-1-triazene (P-NONOate), concanavalin A (ConA), triphosgene, glutathione peroxidase (GPx，from bovine red blood cells) and thioredoxin reductase (TrxR， from rat hepatocytes) were purchased from Sigma-Aldrich Co., Ltd. (Saint Louis, MO, USA). 2-(4-(2-hydroxyethyl)piperazin-1-yl)ethanesulfonic acid (HEPES), potassium chloride, calcium chloride, sodium chloride, magnesium chloride, sodium nitrite ($NaNO_2$), sodium hypochlorite, hydrogen peroxide ($H_2O_2$), homocysteine (Hcy), sodium sulfide ($Na_2S$), sodium hydrosulfide (NaHS), glutathione (GSH), paraformaldehyde, DL-dithiothreitol (DTT) and cysteine (Cys) were purchased from Shanghai Aladdin Biochemical Technology Co., Ltd. (Shanghai, China). Dulbecco's modified eagle medium (DMEM), fetal bovine serum (FBS), roswell park memorial institute-1640 (RPMI-1640), methyl thiazolyl tetrazolium, phosphate buffered saline (PBS), trypsin-EDTA solution were purchased from Beijing Suolaibao Biology Co., Ltd. (Beijing, China). Tert-butyl glycinate and N-9-fluorenylmethoxycarbonyl-Se-4-methoxybenzylselenocysteine was purchased from Shanghai Jizhi Biochemical Technology Co., Ltd. (Shanghai, China). INF-γ was purchased from Shanghai Youningwei Biotechnology Co., Ltd. (Shanghai, China). Isoflurane was purchased from Shenzhen Ruiwode Life Technology Co., Ltd. (Shenzhen, China). Aspartate transaminase (AST) and alanine transaminase (ALT) test kits were purchased from Zhongsheng Beikong Biotechnology Co., Ltd. (Beijing, China). Cysteine hydropersulfide (Cys-SSH) was produced by the reaction of Cys with

NaHS in the presence of P-NONOate [1]. $ONOO^-$ donor is 3-morpholinosydnonimine hydrochloride (SIN-1, 200 μmol/mL) [2]. $NO_2^-$, $K^+$, $Ca^{2+}$, $Na^+$, $Mg^{2+}$ and $ClO^-$ were from $NaNO_2$, potassium chloride, calcium chloride, sodium chloride, magnesium chloride and sodium hypochlorite, respectively. Selenocysteine (Sec) was produced by the reaction of $(CysSe)_2$ with DTT [3]. $O_2^{\bullet -}$ was generated by the enzymatic reaction of xanthine/xanthineoxidase [4]. NO was generated by the 3-(aminopropyl)-1-hydroxy-3-isopropyl-2-oxo-1-triazene [5]. The water for the experiments was ultrapure with a resistance of ≥18.2 MΩ.

## Instruments

The $^1H$ nuclear magnetic resonance ($^1H$ NMR) and $^{13}C$ NMR spectra were obtained using a 500 MHz Avance II DRX-500 NMR spectrometer (Bruker BioSpin GmbH, Rheinstetten, Germany). The mass spectra were obtained from an exactive ultra-high resolution liquid chromatography-mass spectrometry (LC-MS) system (Thermo Fisher Scientific Inc., Waltham, MA, USA). The Multiskan Mk3 microplate reader (photometer) was employed for enzyme labeling assays (Thermo Fisher Scientific Inc., Waltham, MA, USA). The Cary 60 UV−vis spectrophotometer was used for recording the absorption spectrum (Agilent Technologies Inc., Santa Clara, CA, USA). All optoacoustic measurements were performed by using a MSOT inVision 256-TF multispectral photoacoustic imaging system was (iThera Medical GmbH, Munich, Germany).

## Synthesis and characterization of the probe

### Synthetic route of the compound Cy:

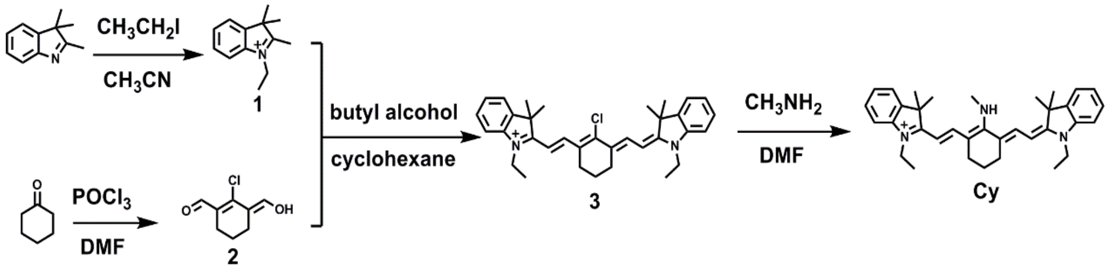


Synthesis of compound 1: 2,3,3-trimethylindolenine (3.18 g, 20 mmol), iodoethane (4.67 g, 30 mmol) and 15 mL of acetonitrile were added into a 50-mL flask. The mixture was heated to 55 °C, and the reaction was performed for 2 h. After cooling to room temperature, the crude product was collected by filtration and purified by

recrystallization in ethanol to afford compound 1 as white crystal (2.73 g, yield 73%). LC-MS (ESI, positive mode): m/z calcd 188.14338, found 188.14265 for $[M]^+$.

Synthesis of compound 2: a mixture of phosphorus oxitrichloride (18.64 mL, 200 mmol) and dichloromethane (17.5 mL) was slowly added into the mixture of dichloromethane (20 mL) and N,N-dimethylformamide (20 mL) under ice bath condition, and then cyclohexanone (5.29 mL, 51 mmol) was added slowly. After removal from the ice bath, the reaction solution was heated to reflux. After stirring the reaction for 4 h, the reaction solution was cooled to room temperature, and then the reaction mixture was poured into 100 g of crushed ice four times. After allowing to stand overnight, the reaction product solution was filtered, and washed with frozen acetone to ultimately obtain a bright yellow crystal (5.39 g, yield 61%).

Synthesis of compound 3: a mixed solution (150 mL) of n-butanol and cyclohexane (7:3, v/v) was added to a 250-mL flask, then compound 1 (1.50 g, 8 mmol) and compound 2 (0.69 g, 4 mmol) were added into the above flask under a nitrogen atmosphere. The mixture was heated to 117 °C, and subjected to reflux reaction for 6 h. Then, the n-butanol and cyclohexane were removed by rotating evaporation. A mixed solution of DCM/MeOH (100:1), v/v) was used as eluate to purify the crude product by column chromatography to give compound 3 as gold-green crystal (1.35 g, yield 66%). Liquid chromatography-mass spectrometry (LC-MS; ESI, positive mode): m/z calcd 511.28745, found 511.28610 for $[M]^+$.

Synthesis of compound Cy: compound 3 (0.194 g, 0.38 mmol) and methylamine hydrochloride (0.027 g, 0.40 mmol) were added to a 50-mL round-bottom flask. Then, 15 mL of dimethylformamide (DMF) and 100 μL of triethylamine were added to the flask under a nitrogen atmosphere. After stirring at 70 °C for 1 h, the mixed solvent was removed by the vacuum distillation method. The crude product was purified by column chromatography using DCM/MeOH (70:1, v/v) as eluent to afford compound Cy as a blue crystal (0.08 g, yield 40%). LC-MS (ESI, positive mode): m/z calcd 506.35297, found 506.35144 for $[M]^+$. $^1$H NMR (400 MHz, $CD_2Cl_2$): δ 8.75 (s, 1H), 7.58 (d, $J$ = 12.7 Hz, 2H), 7.18 (dt, $J$ = 7.3, 3.8 Hz, 4H), 6.94 (t, $J$ = 7.4 Hz, 2H), 6.78 (d, $J$ = 8.1 Hz, 2H), 5.51 (d, $J$ = 12.4 Hz, 2H), 3.76 (d, $J$ = 5.2 Hz, 4H), 3.45 (d, $J$ = 5.3 Hz, 3H), 2.45 (t, $J$ = 6.2 Hz, 4H), 1.75 – 1.70 (m, 2H), 1.59 (s, 12H), 1.25 – 1.21 (m, 6H). $^{13}$C NMR (100 MHz, $CD_2Cl_2$): δ 170.5, 166.3, 143.3, 140.7, 137.0, 128.5, 122.6, 122.4, 108.4, 93.6, 47.9, 37.8, 29.3, 26.7, 21.6, 11.6.

**Synthetic route of compound Cy-1:**

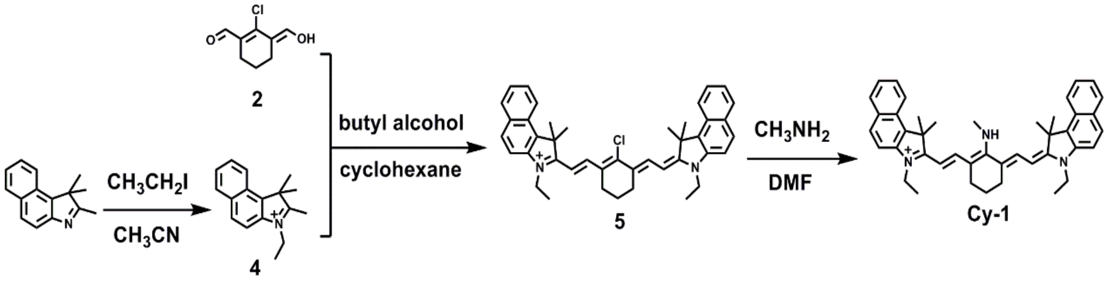


Synthesis of compound 4: the synthesis method was similar to that of compound 1. Using 1,1,2- trimethyl-1H-benz[e]indole (4.18 g, 20 mmol) as raw material, we obtained a blue-white crystal (2.91 g, yield 61%). LC-MS (ESI, positive mode, m/z) calcd. 238.15903, found 238.15831 for $[M]^{+}$.

Synthesis of compound 5: the synthesis method was similar to that of compound 3. Using compound 4 (1.90 g, 8 mmol) as raw material and DCM/MeOH (80:1, v/v) as eluate, we obtained a dark green-brown crystal (1.93 g, yield 53%). LC-MS (ESI, positive mode): m/z calcd. 611.31875, found 611.31677 for $[M]^{+}$.

Synthesis of compound Cy-1: the synthesis method was similar to that of compound Cy. Using compound 5 (0.23 g, 0.38 mmol) as raw material and DCM/MeOH (65:1, v/v) as eluent, we obtained a blue crystal (0.09 g, yield 38%). LC-MS (ESI, positive mode): m/z calcd 606.38427, found 606.38281 for $[M]^{+}$. $^{1}$H NMR (400 MHz, $CD_2Cl_2$): δ 8.68 (s, 1H), 8.01 (d, *J* = 8.6 Hz, 2H), 7.76 (d, *J* = 8.5 Hz, 6H), 7.43 (d, *J* = 8.8 Hz, 2H), 7.26 (t, *J* = 7.5 Hz, 2H), 7.13 (d, *J* = 8.8 Hz, 2H), 5.56 (d, *J* = 12.8 Hz, 2H), 3.89 (s, 4H), 3.51 (d, *J* = 4.8 Hz, 3H), 2.49 (t, *J* = 6.2 Hz, 4H), 1.91 (s, 12H), 1.79 – 1.74 (m, 2H), 1.29 (t, *J* = 7.0 Hz, 6H). $^{13}$C NMR (100 MHz, $CD_2Cl_2$): δ 170.1, 168.2, 136.9, 131.2, 130.3, 130.3, 129.2, 127.6, 124.0, 122.5, 110.1, 93.3, 49.9, 37.9, 28.8, 26.7, 21.8, 12.0.

**Synthetic route of compound Cy-2:**

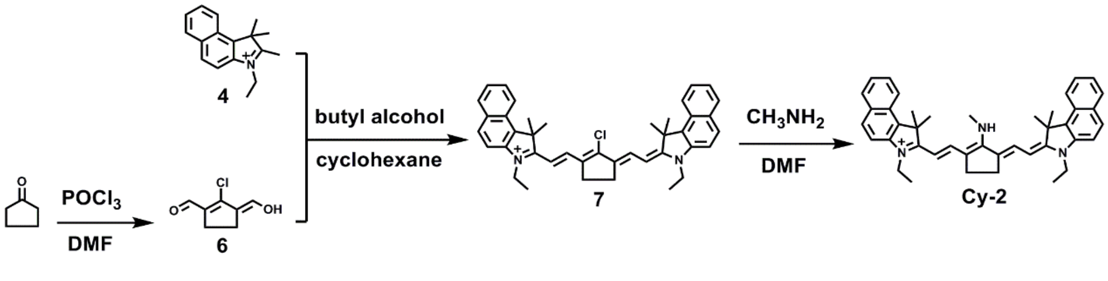


Synthesis of compound 6: the synthesis method was similar to that of compound 2. Using cyclopentanone (4.51 mL, 51 mmol) as raw material, we obtained a bright yellow crystal (3.48 g, yield 43%). The product was not characterized and directly used in the

next reaction.

Synthesis of compound 7: the synthesis method was similar to that of compound 3. Using compound 4 (1.90 g, 8 mmol) and compound 6 (0.63 g, 4 mmol) as raw materials, DCM/MeOH (80:1, v/v) as eluate, we obtained a red-brown crystal (1.17 g, yield 49%). LC-MS (ESI, positive mode): m/z calcd 597.30310, found 597.30109 for $[M]^+$. $^1H$ NMR (400 MHz, $CD_2Cl_2$): δ 8.13 (d, $J$ = 8.0 Hz, 2H), 7.98 (d, $J$ = 4.0 Hz, 2H), 7.94 (d, $J$ = 9.7 Hz, 5H), 7.61 (dd, $J$ = 8.3, 4.1 Hz, 2H), 7.52 – 7.41 (m, 5H), 6.04 (d, $J$ = 14.1 Hz, 2H), 4.26 (t, $J$ = 7.3 Hz, 4H), 3.03 (s, 4H), 2.01 (s, 3H), 1.98 (s, 9H), 1.48 (t, $J$ = 7.2 Hz, 6H). $^{13}C$ NMR (100 MHz, $CD_2Cl_2$): δ 172.0, 151.4, 144.0, 141.5, 139.4, 137.2, 136.2, 133.9, 131.9, 130.8, 130.1, 128.2, 127.8, 125.1, 122.2, 110.8, 102.0, 101.4, 51.1, 40.1, 27.3, 27.3, 26.7, 12.5.

Synthesis of compound Cy-2: the synthesis method was similar to that of compound Cy. The compound 7 (0.227 g, 0.38 mmol) was used as the starting material, the reaction temperature and time were 40 °C and 15 min, respectively. DCM/MeOH (65:1, v/v) was used as the eluent to obtain a blue crystal (0.18 g, yield 79%). LC-MS (ESI, positive mode): m/z calcd 592.36862, found 592.36743 for $[M]^+$. $^1H$ NMR (400 MHz, $CD_2Cl_2$): δ 8.92 (d, $J$ = 5.4 Hz, 1H), 8.25 (d, $J$ = 12.8 Hz, 2H), 8.10 (d, $J$ = 8.5 Hz, 2H), 7.87 – 7.84 (m, 3H), 7.83 (s, 1H), 7.50 (t, $J$ = 7.6 Hz, 2H), 7.34 (t, $J$ = 7.5 Hz, 2H), 7.22 (d, $J$ = 8.7 Hz, 2H), 5.55 (d, $J$ = 12.8 Hz, 2H), 3.96 (d, $J$ = 7.2 Hz, 4H), 3.66 (d, $J$ = 5.4 Hz, 3H), 2.81 (d, $J$ = 4.0 Hz, 4H), 2.05 (s, 12H), 1.37 (dd, $J$ = 9.7, 4.3 Hz, 6H). $^{13}C$ NMR (100 MHz, $CD_2Cl_2$): δ 168.6, 165.6, 162.8, 140.5, 135.2, 131.9, 131.3, 130.3, 130.2, 129.7, 129.0, 127.6, 127.5, 126.9, 124.0, 122.5, 116.1, 110.1, 95.7, 50.0, 38.5, 34.5, 28.0, 26.8, 12.0.

**Synthesis of compound Cy-3:**

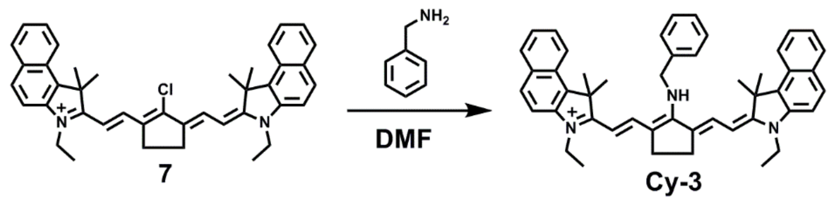


The compound 7 (0.23 g, 0.38 mmol), benzylamine (44 μL, 0.40 mmol) and 15 mL DMF were added to a 50-mL round-bottom flask under a nitrogen atmosphere. After stirring the reaction at 40 °C for 30 min, the mixed solvent was removed by the vacuum distillation method. Then, the crude product was purified by column chromatography using DCM/MeOH (60:1, v/v) as eluent to afford compound Cy-3 as a blue crystal

(0.17 g, yield 68%). LC-MS (ESI, positive mode): m/z calcd 668.39993, found 668.40039 for $[M]^+$. $^1$H NMR (400 MHz, $CD_2Cl_2$): δ 8.92 (t, *J* = 6.4 Hz, 1H), 7.99 – 7.87 (m, 4H), 7.75 (t, *J* = 7.8 Hz, 4H), 7.41 (t, *J* = 7.4 Hz, 4H), 7.36 (d, *J* = 7.2 Hz, 2H), 7.30 – 7.23 (m, 3H), 7.12 (d, *J* = 8.8 Hz, 2H), 5.47 (d, *J* = 12.8 Hz, 2H), 5.18 (d, *J* = 7.0 Hz, 2H), 3.87 (d, *J* = 6.9 Hz, 4H), 2.75 (s, 4H), 1.63 (s, 12H), 1.27 (t, *J* = 7.1 Hz, 6H). $^{13}$C NMR (100 MHz, $CD_2Cl_2$): δ 168.6, 165.5, 140.0, 137.2, 131.0, 129.9, 129.8, 129.3, 128.7, 127.7, 127.3, 126.3, 123.8, 122.2, 109.8, 95.8, 58.2, 49.6, 49.1, 38.2, 27.3, 26.8, 18.4, 11.7.

**Synthesis of compound APSel probe:**

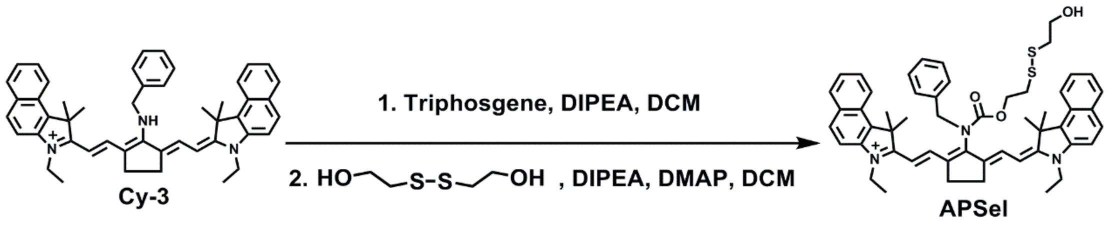


The synthesis method was similar to that reported previously [6]. Using compound Cy-3 (0.07 g, 0.10 mmol) as raw material, we obtained a red-brown crystal (0.05 g, yield 58%).

## Absorption spectrum and *in vitro* PA response of APSel to Sec

The APSel was dissolved in DMSO to prepare the stock solution (1 mM), and was stored at 4 °C. HEPES buffer solution (10 mM, pH 7.4) was mixed with APSel stock solution to obtain a APSel test solution (containing 20% DMSO, v/v). The reaction time for APSel with Sec was set to 5 min in subsequent experiments, and all experiments were performed at 37 ºC. In the experiments to assess the time dependence of the probe response, the absorption spectra were recorded every 30 s after mixing the 5 μM APSel with the 10 μM Sec. For the evaluation of the stability of the probe, the absorption spectra of APSel and the reaction mixture of APSel in the presence of Sec at different time periods (0, 2, 4, 7, 12 h) were recorded.

For *in vitro* PA tests, the sample was transferred to a transparent suction 3-mm diameter tube, and embedded in the agar phantom. The agar phantom was fixed to the bracket and placed in water for testing. The PA imaging signals at 690 and 860 nm were recorded, and the PA intensity ratio $PA_{690}/PA_{860}$ was used to quantify the concentration of Sec. The detection limit was determined according to 3σ/κ (where σ is the standard deviation for the blank APSel solution in 20 measurements, κ is the slope of the linear

regression curve for $PA_{690}/PA_{860}$ to the Sec concentration. In the selectivity teste, PA imaging was performed after the APSel was mixed with different analytes for 20 min.

## Cell culture

The HepG2 and HL-7702 cell lines were purchased from the Cell Bank of the Typical Culture Storage Committee of the Chinese Academy of Sciences / Cell Resource Center of the Shanghai Institute of Life Sciences of the Chinese Academy of Sciences (Shanghai, China). HepG2 cells were cultured in DMEM (with 10% FBS). HL-7702 cells were cultured in RPMI-1640 (with 10% FBS). The cells were cultured in a cell incubator at 37 °C with a humidified atmosphere of 5% $CO_2$ in air. The medium was replaced every 2 days.

## Cytotoxicity test

The MTT (methyl thiazolyl tetrazolium) assay was used to determine the cytotoxicity of APSel. The cells were seeded on 96-well cell culture plates and incubated at 37 °C in 5% $CO_2$ for 24 h. Then, the APSel solution at different final concentrations of 0, 5,10,15,30 μM was added into the corresponding well in the cell culture plates. The cells were cultured in an incubator at 37 °C with 5% $CO_2$ for 24 h. Subsequently, 15 μL of MTT solution (5 mg/mL) was added to each well, and the plate was incubated for another 4 h at 37 °C with 5% $CO_2$. Then, 150 μL of DMSO was added into each well after removing the medium. Finally, the absorbance of each well was measured at 570 nm wavelength using a Multiskan Mk3 microplate reader.

## PA imaging of cells

The four groups of HL-7702 cells were seeded into the T75 flask, and cultured for 24 h at 37 °C with 5% $CO_2$. The medium in the first and second groups of cells was replaced with fresh medium. The medium in the third group of cells was replaced with a medium containing $Na_2SeO_3$ (5 μM). The medium in the fourth group of cells was replaced with a medium containing INF-γ (60 ng/mL). After culturing for 24 h, the medium from each flask was removed, and the cells were washed three times with PBS (pH 7.4), and then digested with trypsin. The first group of cells was suspended in pure PBS, and the remaining groups of cells were suspended in PBS containing APSel (5 μM, 2% DMSO, v/v). Then, the cells in each group were continued to culture for 1 h at room temperature. Finally, each group of cells was transferred to a 300-μL centrifuge tube and centrifuged at 1,500 rpm and 4 °C for 7 min to obtain the cell pellet. The

centrifuge tube was fixed to the support, and PA imaging was performed on the multispectral photoacoustic tomography imaging system in water at 25 °C with excitation at 690 nm and 860 nm.

## Animal model

Twelve-week-old male BALB/c mice (24-27 g) were purchased from Hunan SJA Laboratory Animal Co., Ltd. (Changsha, China). All animals were given food and water *ad libitum*, and kept in a sterile environment at room temperature. All animal experiments were approved (No.20150325-XC) by the Animal Ethics Committee of Guangxi Normal University, and were conducted under the protocols of the Care and Use of Laboratory Animals of the Guangxi Normal University.

## *In vivo* toxicity assessment of APSel probe

Two groups of BALB/c mice were injected with either normal saline (control group) or APSel (0.2 mg/kg, test group) *via* tail vein. After 24 h, the mice were euthanized and the main organs (heart, liver, spleen, lung, kidney) were harvested. Then, tissue samples from these organs were fixed in a 4% paraformaldehyde solution and embedded in paraffin to prepare 3-μm thick sections. The sections were stained with hematoxylin and eosin (H & E) and the images of the biological tissue sections were captured under a microscope.

For the chronic toxicity evaluation, two groups of BALB/c mice were injected with normal saline or APSel (0.1, 0.2 mg/kg) *via* tail vein. After 7 days, the blood samples were collected from the eyeball of mice (about 0.8 mL) and routine blood analysis was performed directly on a blood analyzer.

For the assessment of systemic toxicity, two groups of BALB/c mice were injected with either normal saline (control group) or APSel (0.2 mg/kg) *via* tail vein. Then, the body weight of the mice was measured daily for 14 days and checked for clinically relevant abnormalities. When the mice were unable to eat, injured or died, the experiment on the mice was suspended.

## PA imaging of exogenous Sec *in vivo*

The mice were anesthetized by isoflurane (2%), and the hair on their back was shaved. Then, 100 μL of 15 μM APSel solution was injected subcutaneously at both sides of each mouse back into the A and B ROIs. The mice were then coated with ultrasound gel, and were fitted with a breathing mask with 2% isoflurane anesthesia.

Then, the mice were wrapped with a film and placed in water at 34 °C for PA imaging (with 690 and 860 nm wavelength excitation). After that, the mice were further injected with 100 μL of 0.9% of sterile saline in ROI A, and with 100 μL of 30 μM of Sec in ROI B. After 30 min, the PA imaging PA was performed.

## PA imaging of liver from AIH disease model mice

Male BALB/c mice (24-27 g) were randomly divided into five groups. The first and second groups of mice were injected with normal saline (100 μL) *via* tail vein. The remaining groups of mice were injected with normal saline (100 μL) containing Con A (15 mg/kg) *via* the tail vein. After 10 h, the third group was injected with normal saline (150 μL) containing APSel (0.1 mg/kg, 5% DMSO, v/v) *via* tail vein. After 1 h, the mice were euthanized, and their liver was dissected. The liver was photographed and used for PA imaging (5 nm steps, using an excitation in the wavelength range of 680-960 nm), and then fixed in a 4% paraformaldehyde solution. After 24 h, the first, second and fourth groups of mice were subjected to the same procedure as the third group, but the first group of mice were injected with saline without APSel. After 72 h, the fifth group of mice was also subjected to the same procedure as the third group.

## Distribution of probe in mouse organs

Two groups of BALB/c mice were injected with either normal saline (control group) or APSel (0.1 mg/kg, test group). After 1 h, the mice were euthanized, and their main organs (heart, liver, spleen, lung, kidney) were harvested. The organs were immediately coated with ultrasound gel. Then the organs were wrapped with a film and placed in water at 34 °C for PA imaging (5 nm steps, the excitation was in the wavelength range of 680-960 nm).

## Biochemical analysis of normal and AIH disease model mice

The blood collected from normal and AIH disease model mice was kept static at room temperature for 1.5 h and then centrifuged for 10 min (3,000 rpm, 4 °C). Subsequently, the supernatant was used to determine the content of AST and ALT using the automatic biochemical analyzer. The paraformaldehyde fixed liver was sliced into sections, and then the liver sections were stained by H&E, and images captured under a microscope.

## Data analysis

The *in vivo* and *in vitro* PA intensity were obtained by using the post-processing software ViewMSOT (iThera Medical GmbH) of the MSOT inVision 256-TF multispectral photoacoustic tomography imaging system. The GraphPad Prism v7.04 software (GtraphPad Software Inc., La Jolla, CA, USA) was used for statistical analysis. The student's t-test method was used for all data analysis and data are expressed as the mean ±standard deviation. When the P value <0.05, the difference is considered to be statistically significant.

## SUPPORTING REFERENCES


1 T. Ida, T. Sawa, H. Ihara, Y. Tsuchiya, Y. Watanabe, Y. Kumagai, M. Suematsu, H. Motohashi, S. Fujii, T. Matsunaga, M. Yamamoto, K. Ono, N. O. Devariebaez, M. Xian, J. M. Fukuto, T. Akaike, Reactive cysteine persulfides and S-polythiolation regulate oxidative stress and redox signaling. *Proc. Natl. Acad. Sci. USA.*, 2014, **111**, 7606-7611.

2 R. M. Uppu, Synthesis of peroxynitrite using isoamyl nitrite and hydrogen peroxide in a homogeneous solvent system. *Anal. Biochem.*, 2006, **254**, 165-168.

3 H. Maeda, K. Katayama, H. Matsuno, T. Uno, 3’-(2, 4-dinitrobenzenesulfonyl)-2’, 7’-dimethylfluorescein as a fluorescent probe for selenols. *Angew. Chem., Int. Ed.*, 2006, **45**, 1810-1813.

4 V. Massey, H. Komai, G. Palmer, On the mechanism of inactivation of xanthine oxidase by allopurinol and other pyrazolo [3, 4-d] pyrimidines. *J. Biol. Chem.*, 1970, **245**, 2837-2844.

5 H. Maeda, K. Yamamoto, Y. Nomura, I. Kohno, L. Hafsi, N. Ueda, S. Yoshida, M. Fukuda, Y. Fukuyasu, Y. Yamauchi, N. Itoh, A design of fluorescent probes for superoxide based on a nonredox mechanism. *J. Am. Chem. Soc.*, 2005, **127**, 68-69.

6 X. Han, X. Song, F. Yu, L. Chen, A ratiometric near-infrared fluorescent probe for quantification and evaluation of selenocysteine-protective effects in acute inflammation. *Adv. Funct. Mater.*, 2017, **27**, 1700769.

# TABLE

**Table S1.** Photophysical parameters of APSel and its precursors in DMSO

| Comp. | $\lambda_{max}$ (nm) | $\varepsilon$ ($M^{-1}cm^{-1}$) |
|---|---|---|
| Cy | 615 | 5.1E+04 |
| Cy-1 | 643 | 5.7E+04 |
| Cy-2 | 678 | 9.1E+04 |
| Cy-3 | 696 | 9.4E+04 |
| APSel | 869 | 1.5E+05 |

# FIGURES

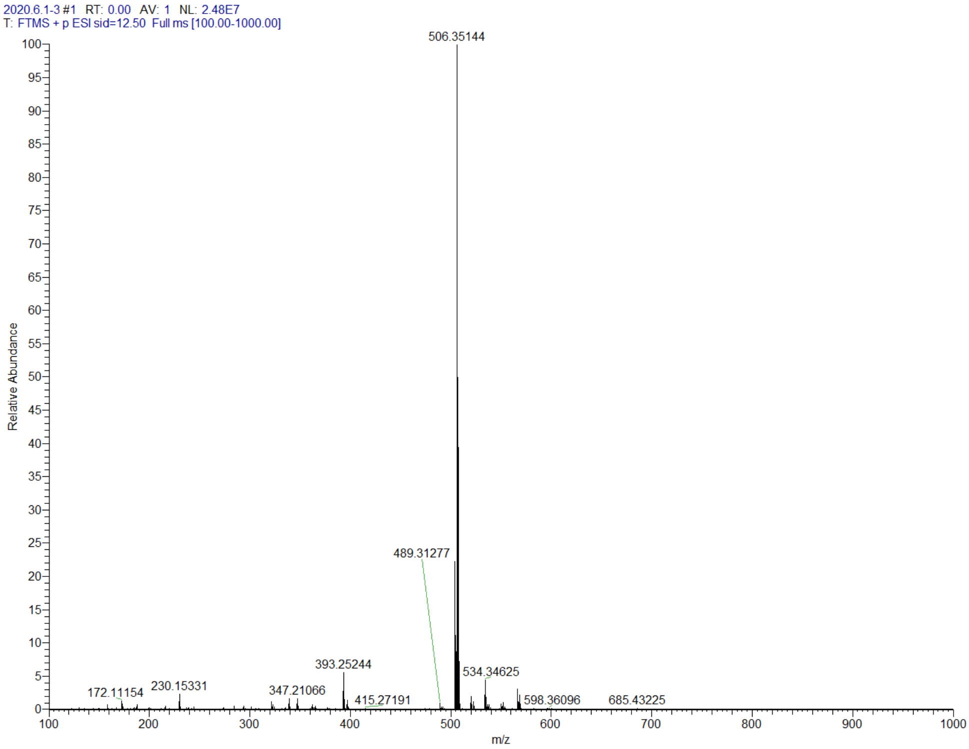


**Fig. S1**. LC-MS spectrum of Cy.

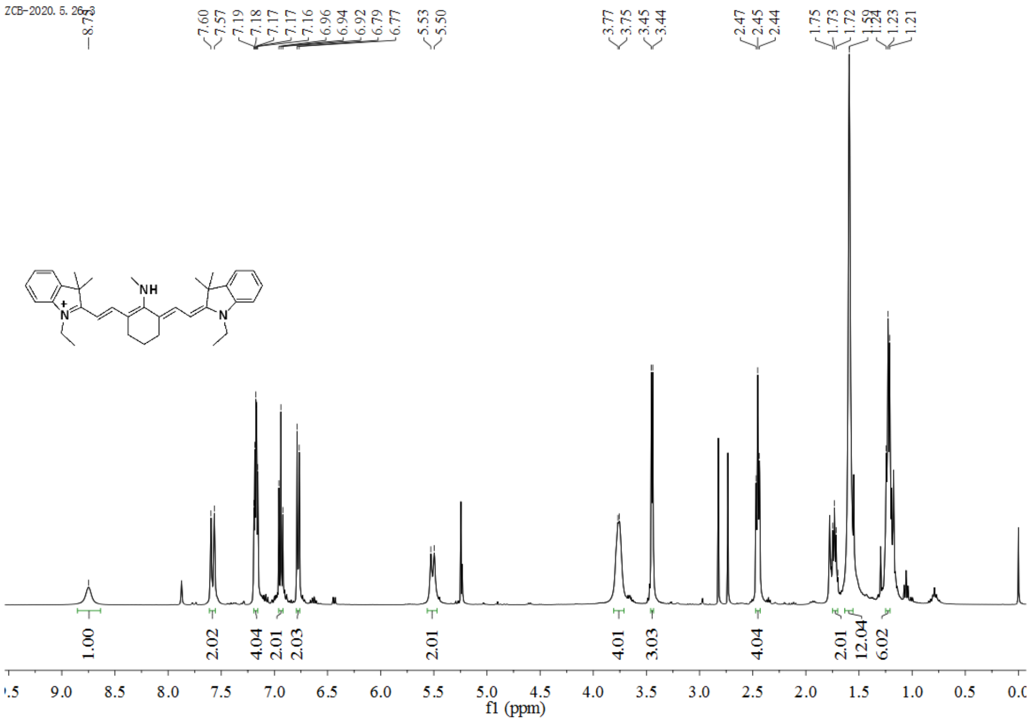


**Fig. S2**. $^{1}$H NMR spectra of Cy in $CD_2Cl_2$.

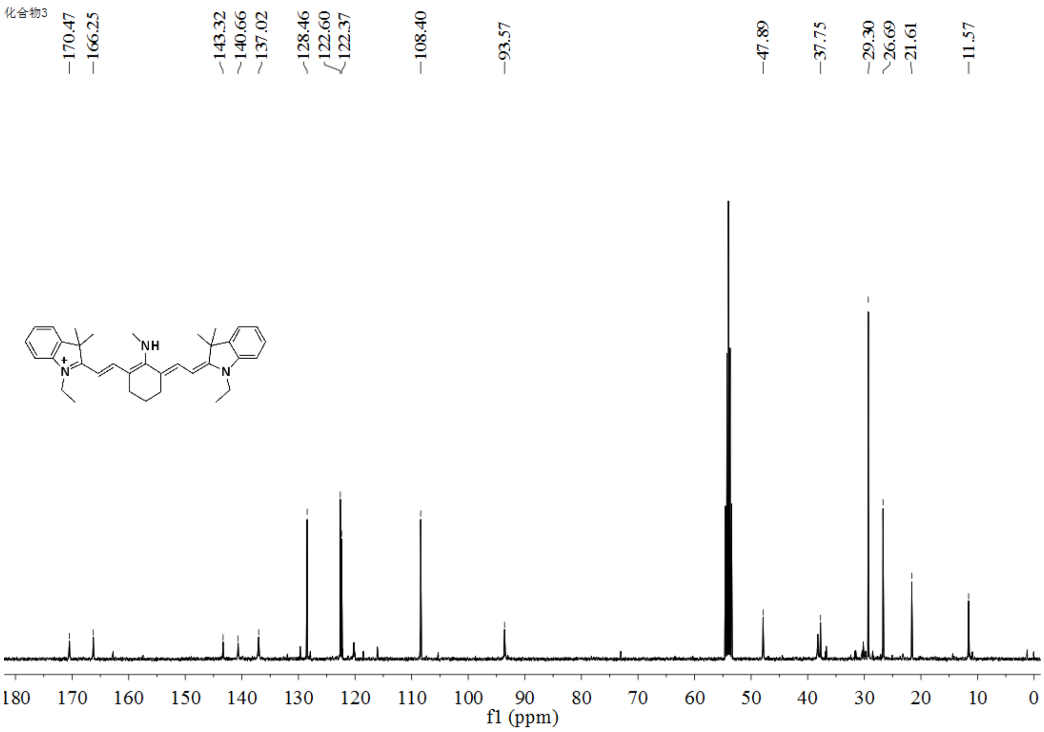


**Fig. S3**. $^{13}C$ NMR spectrum of Cy in $CD_2Cl_2$.

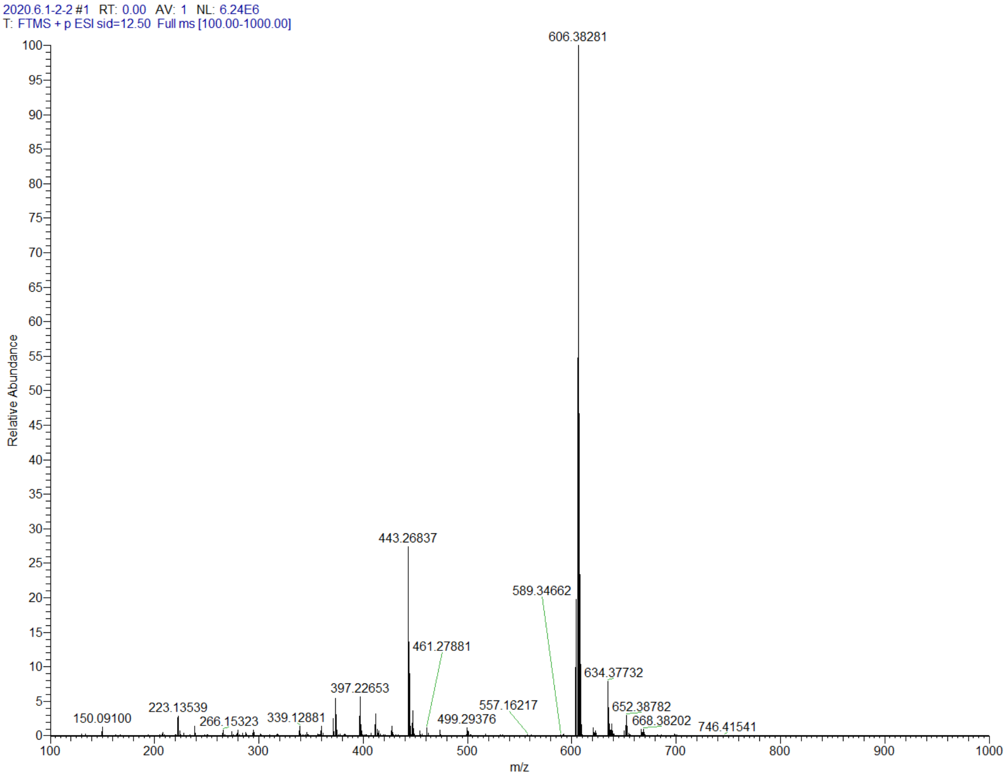


**Fig. S4**. LC-MS spectrum of Cy-1.

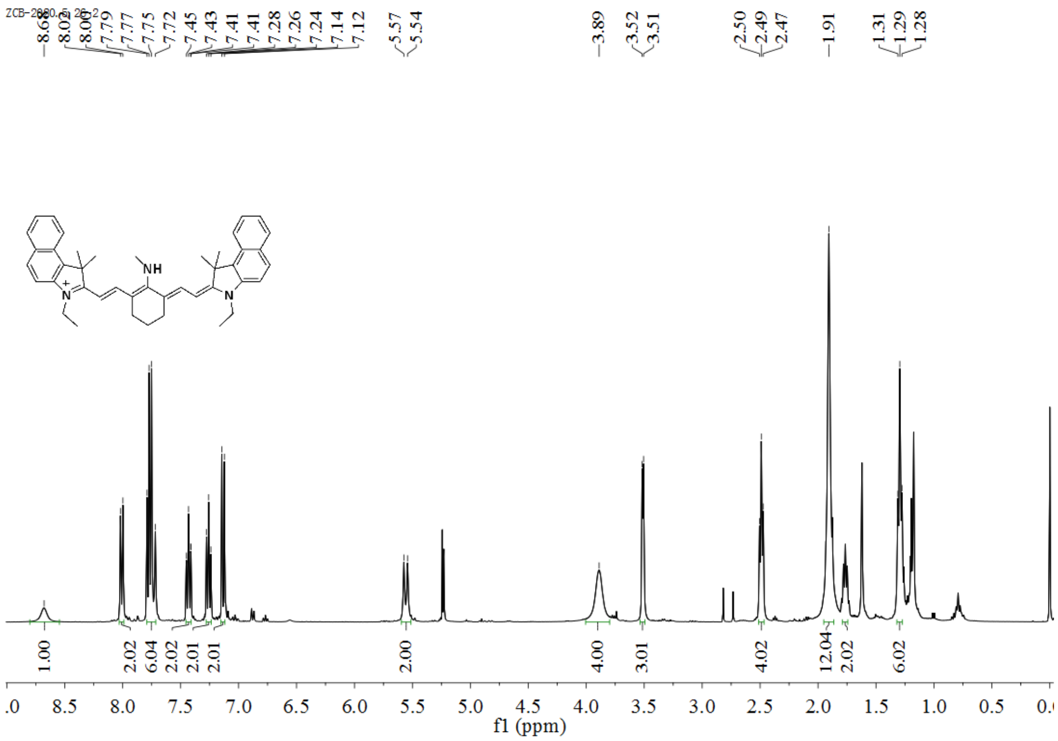


**Fig. S5**. $^{1}$H NMR spectra of Cy-1 in $CD_2Cl_2$.

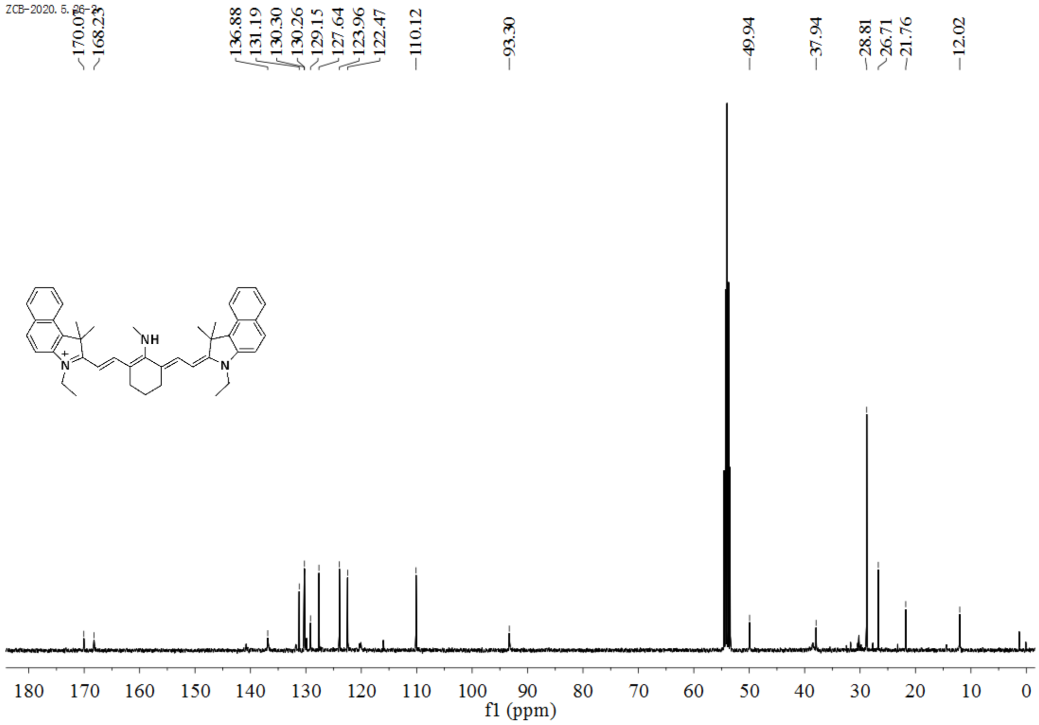


**Fig. S6**. $^{13}$C NMR spectra of Cy-1 in $CD_2Cl_2$.

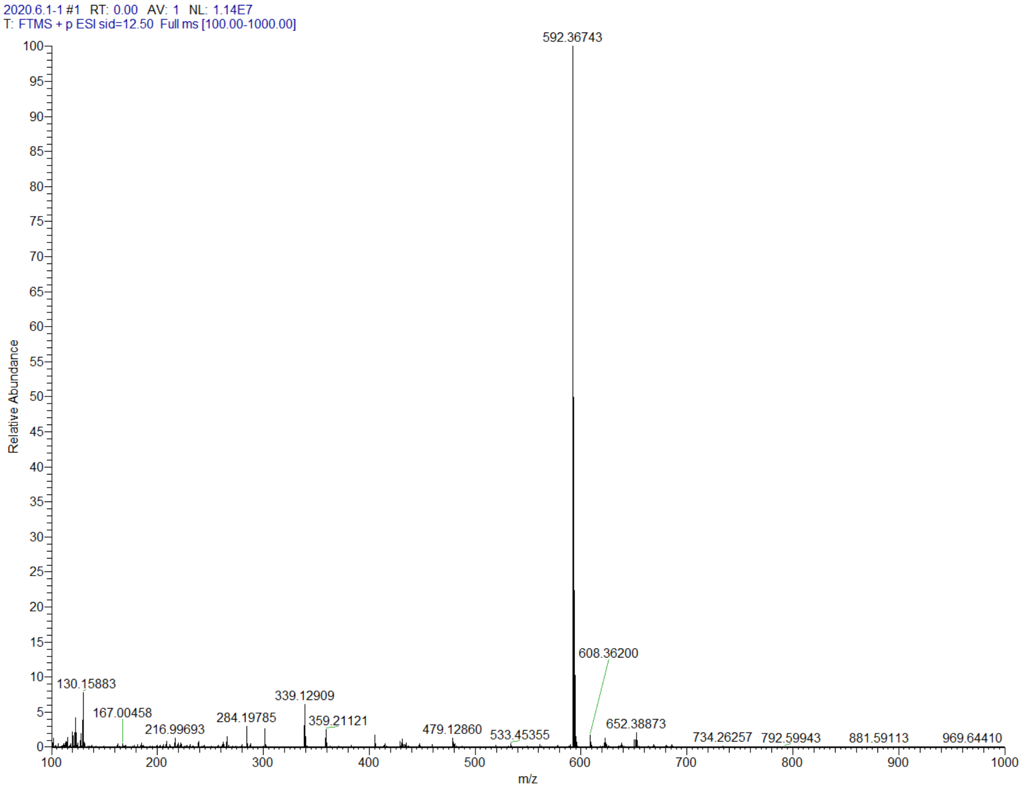


**Fig. S7**. LC-MS spectrum of Cy-2.

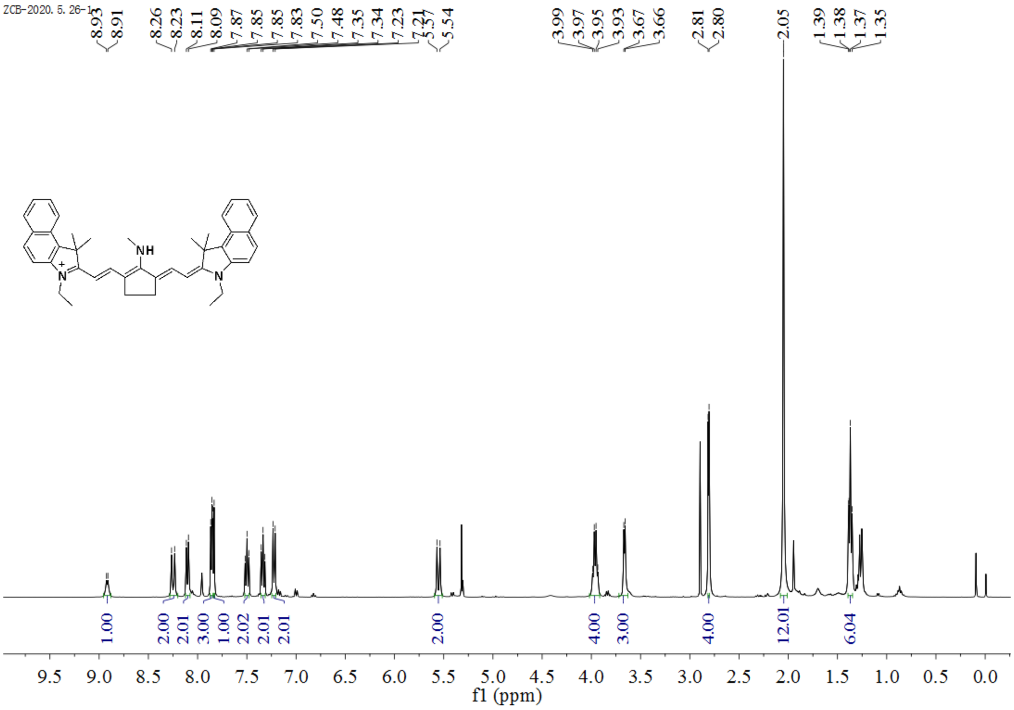


**Fig. S8**. $^{1}$H NMR spectra of Cy-2 in $CD_2Cl_2$.

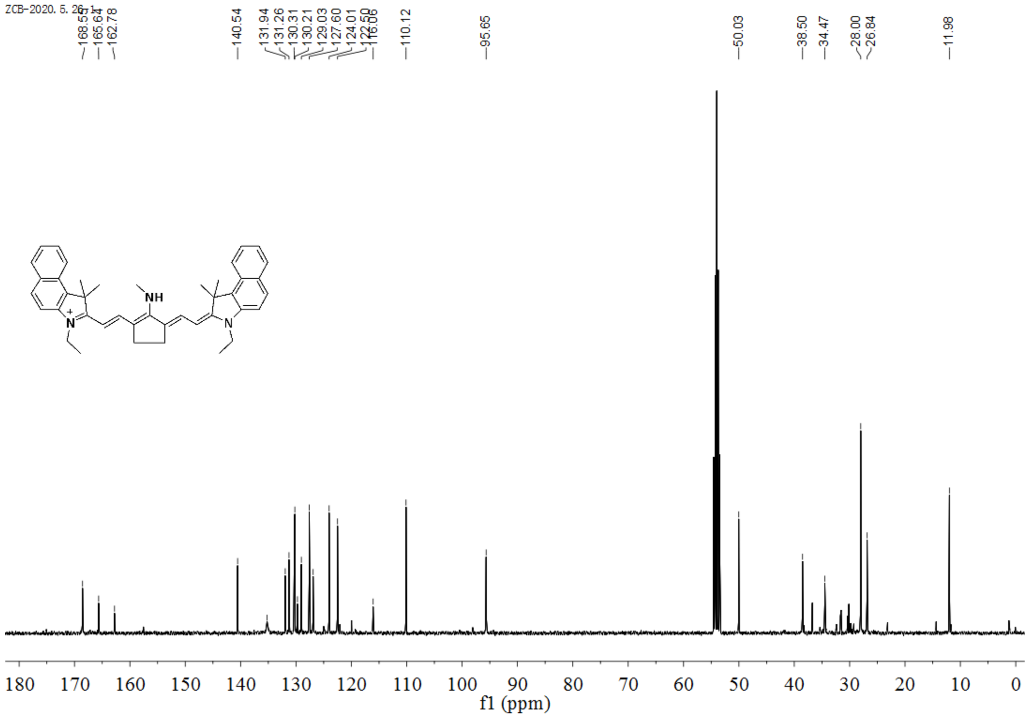

**Fig. S9.** $^{13}$C NMR spectra of Cy-2 in $CD_2Cl_2$.

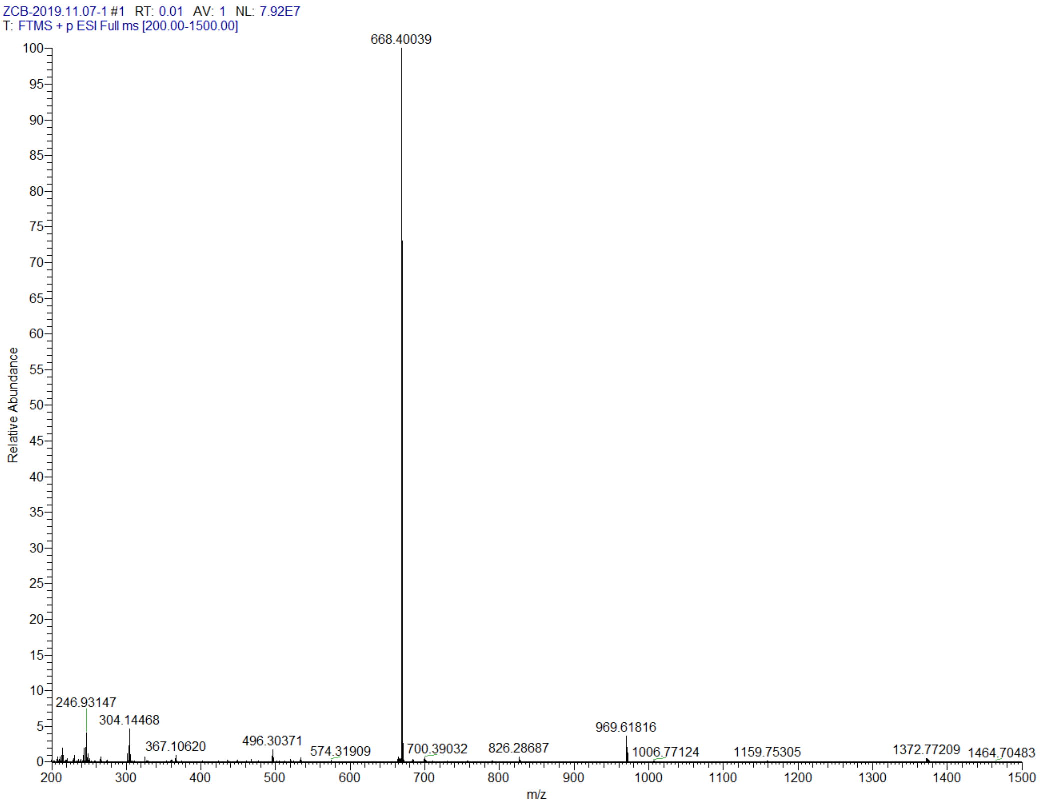

**Fig. S10.** LC-MS spectrum of Cy-3.

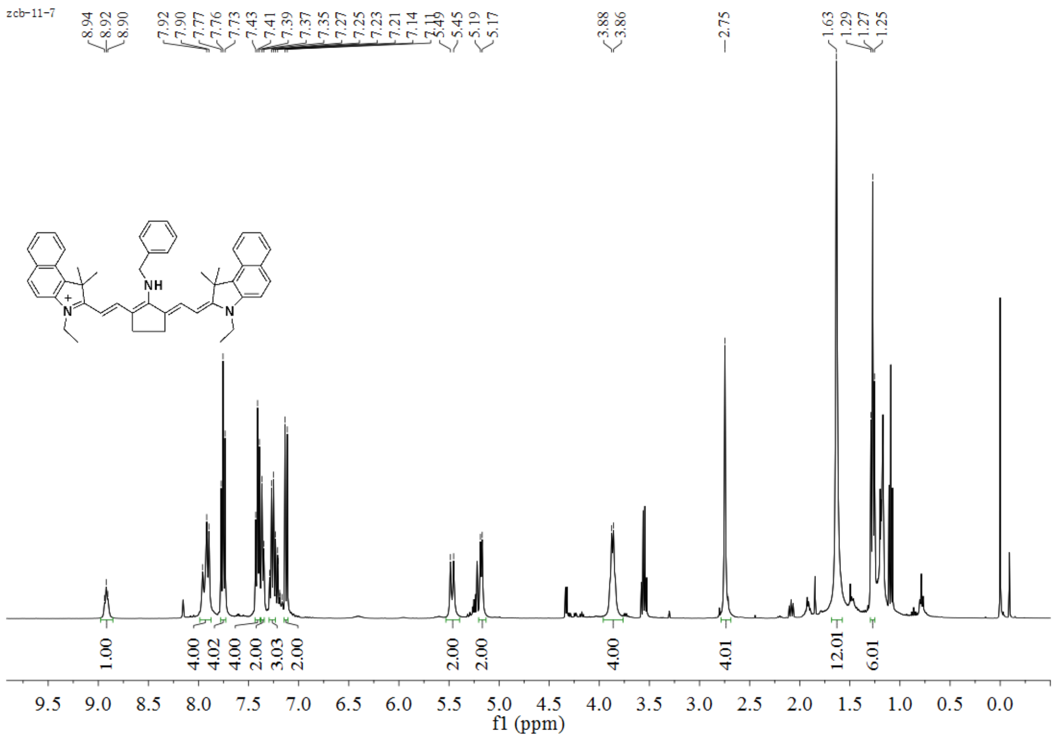


**Fig. S11.** $^{1}H$ NMR spectrum of Cy-3 in $CD_2Cl_2$.

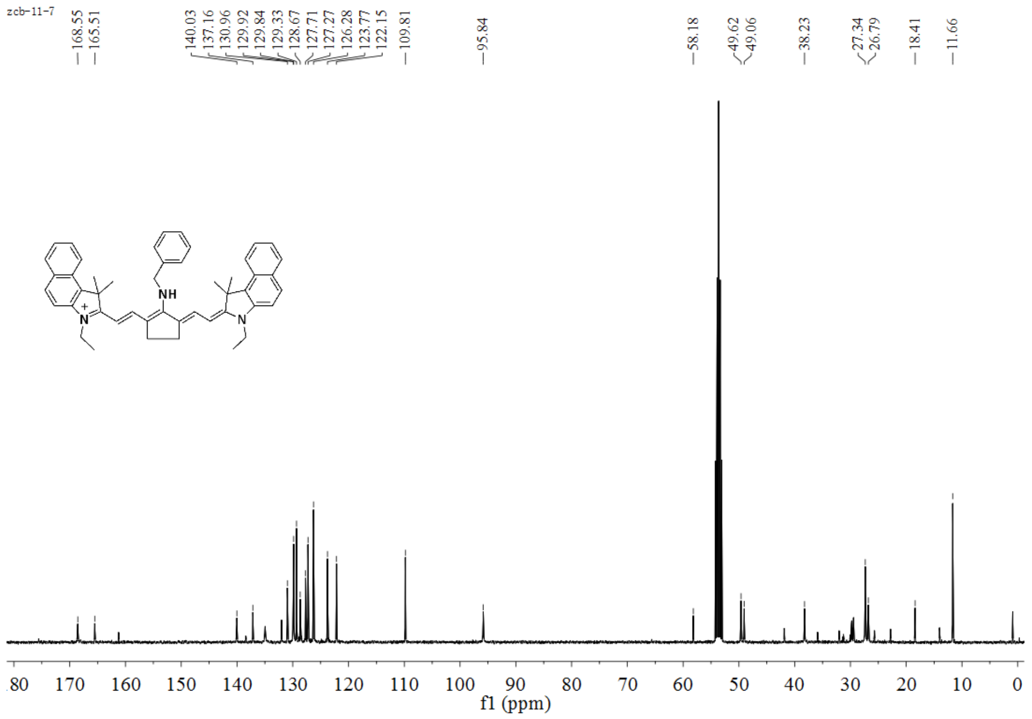


**Fig. S12.** $^{13}C$ NMR spectrum of Cy-3 in $CD_2Cl_2$.

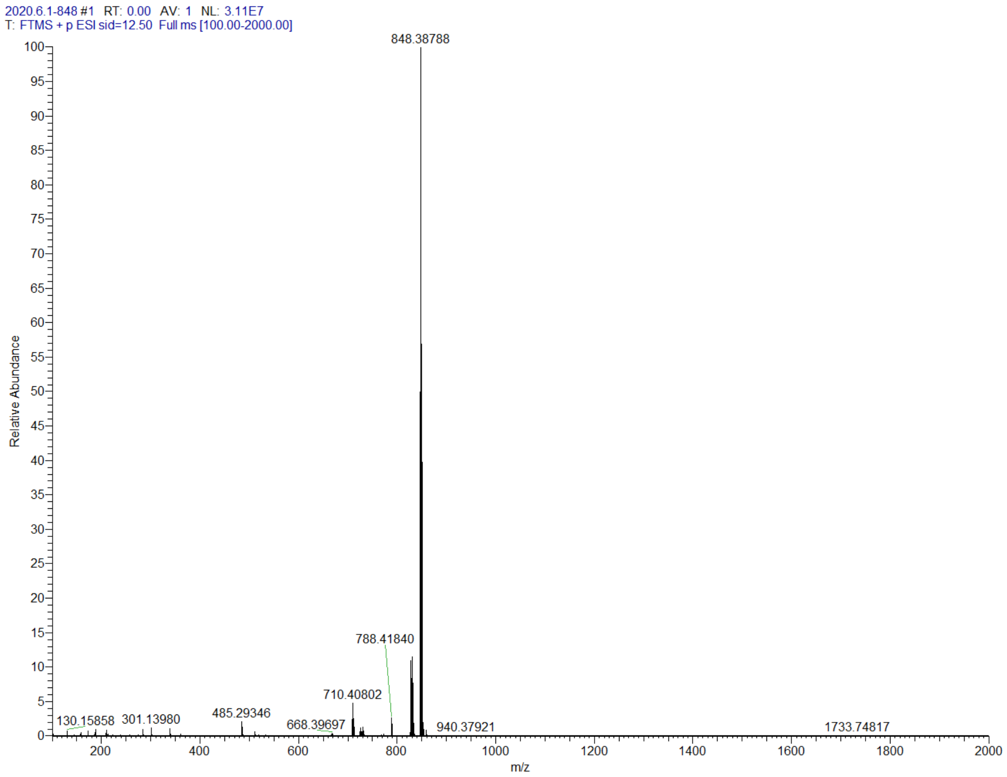


**Fig. S13**. LC-MS spectrum of APSel

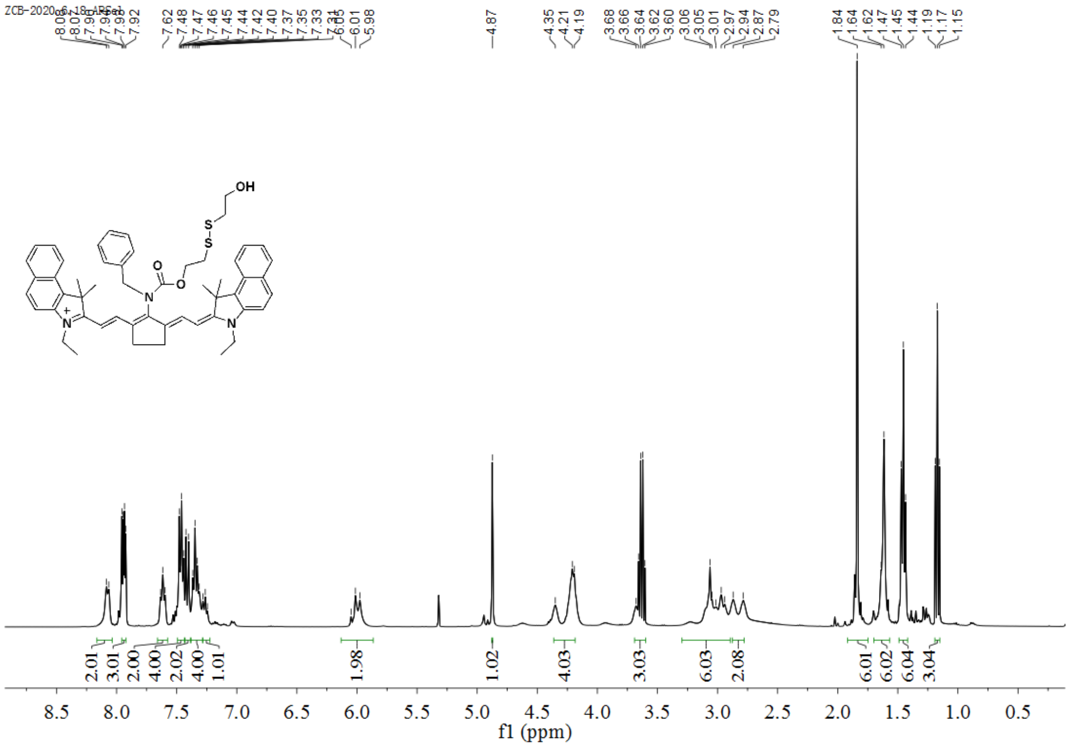


**Fig. S14**. $^{1}$H NMR spectrum of APSel in $CD_2Cl_2$.

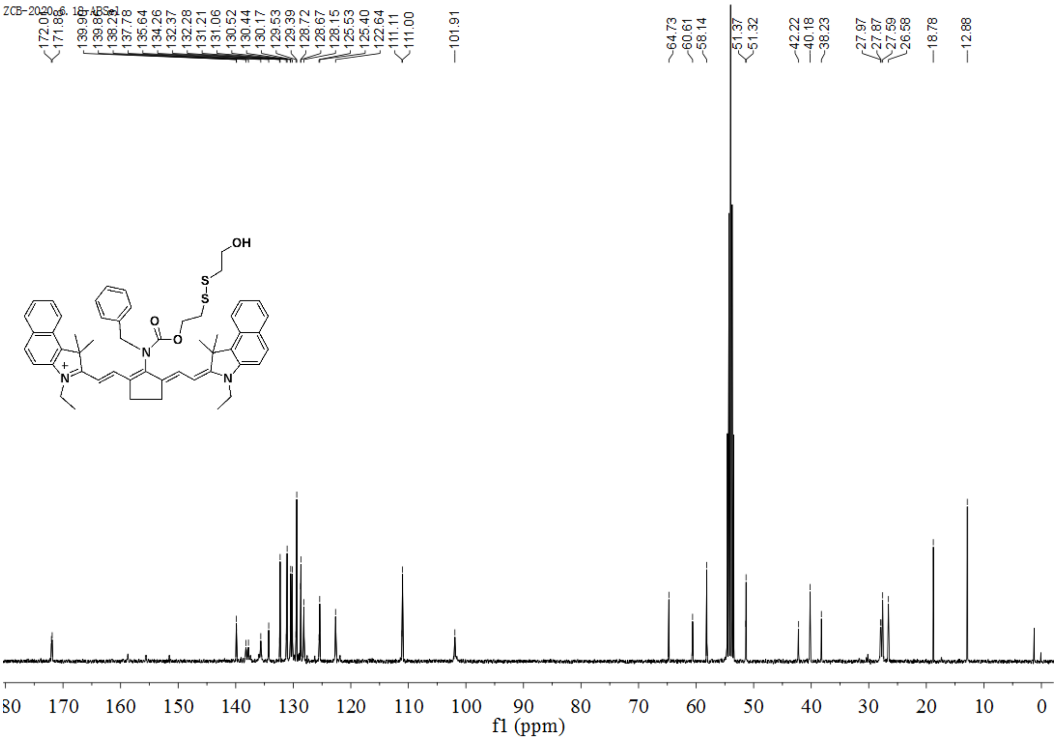


**Fig. S15**. $^{13}C$ NMR spectrum of APSel in $CD_2Cl_2$.

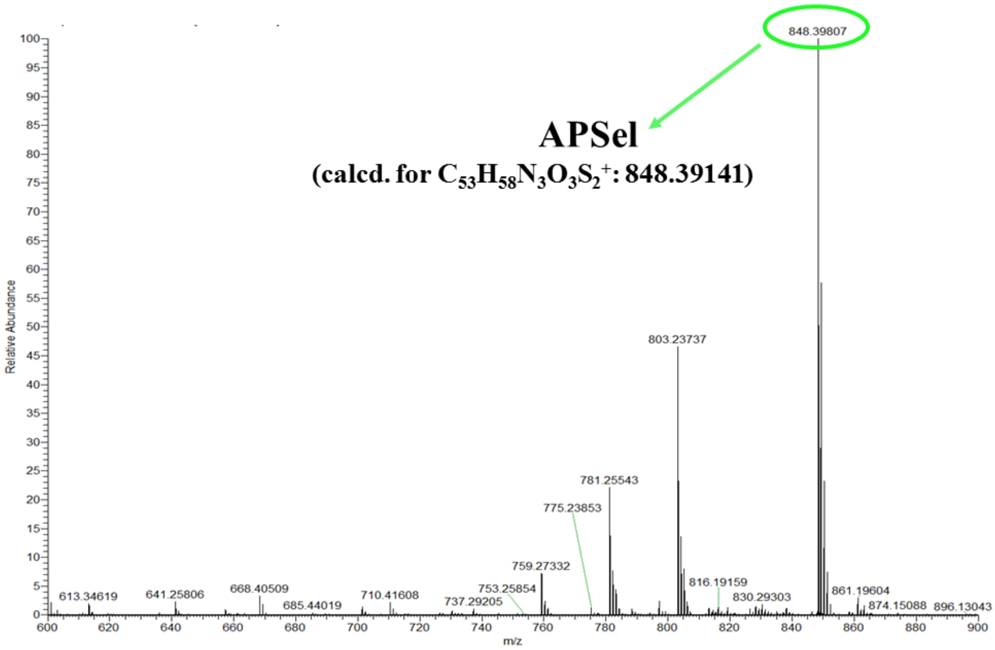


**Fig. S16**. MS spectrum of APSel (5μM) in HEPES buffer solution containing 20% DMSO (v/v).

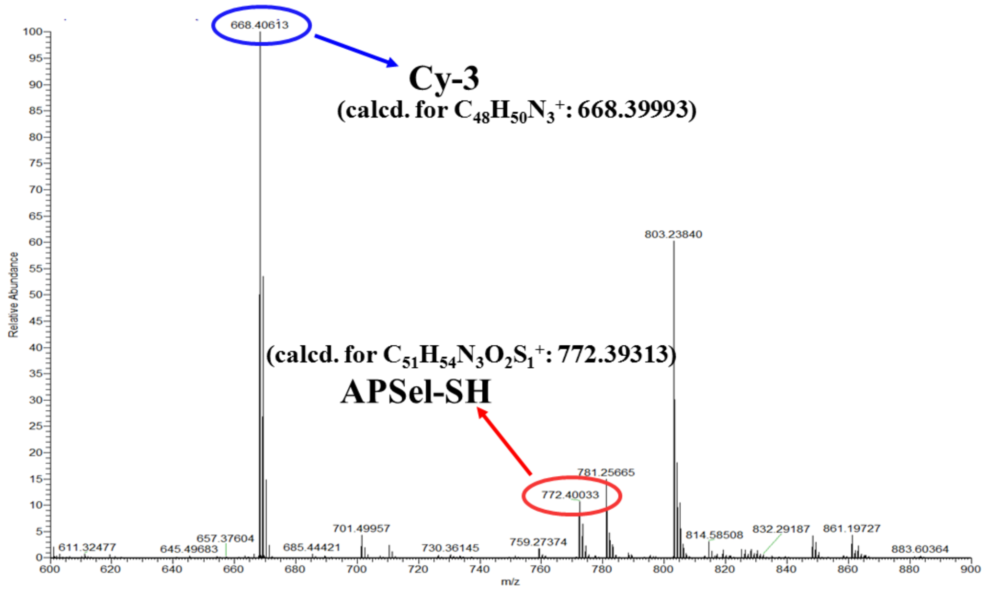


**Fig. S17**. MS spectrum of the reaction product of APSel (5 μM) after adding two equivalent Sec in HEPES buffer solution containing 20% DMSO (v/v).

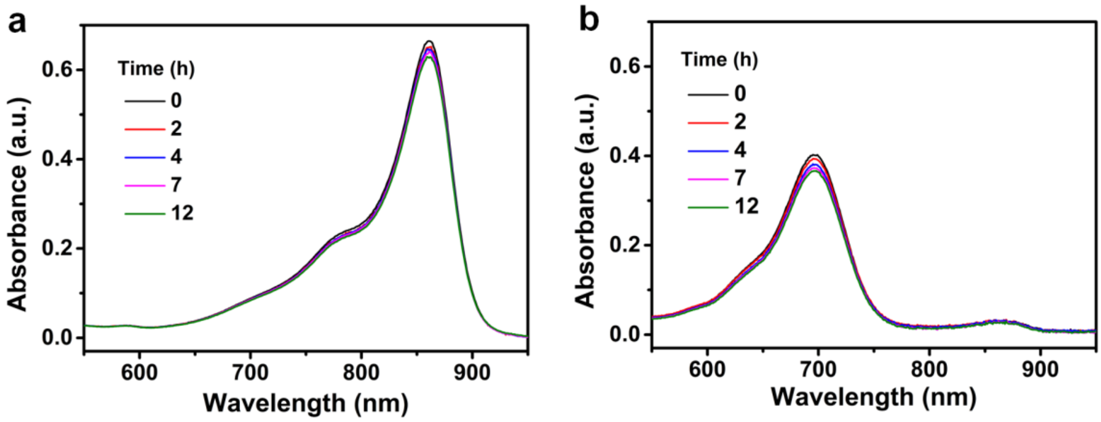


**Fig. S18**. Absorption spectra of APSel (a) and its reaction product with Sec (b) at different time points. The reaction medium was a HEPES buffer solution (10 mM, pH 7.4, 20% DMSO (v/v).

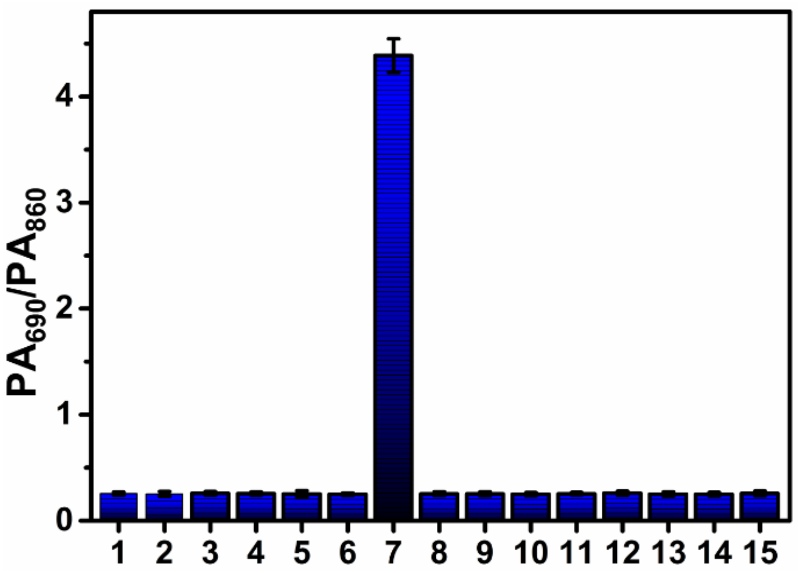


**Fig. S19** $PA_{690}/PA_{860}$ value in the presence of different substances. 1-6: $H_2O_2$, $O_2^{\bullet-}$, $ClO^-$, NO, $ONOO^-$, $NO_2^-$ (100 μM); 7: Sec (10 μM); 8-15: $K^+$, $Ca^{2+}$, $Na^+$, $Mg^{2+}$, Ser, Ala, His, Glu (1 mM).

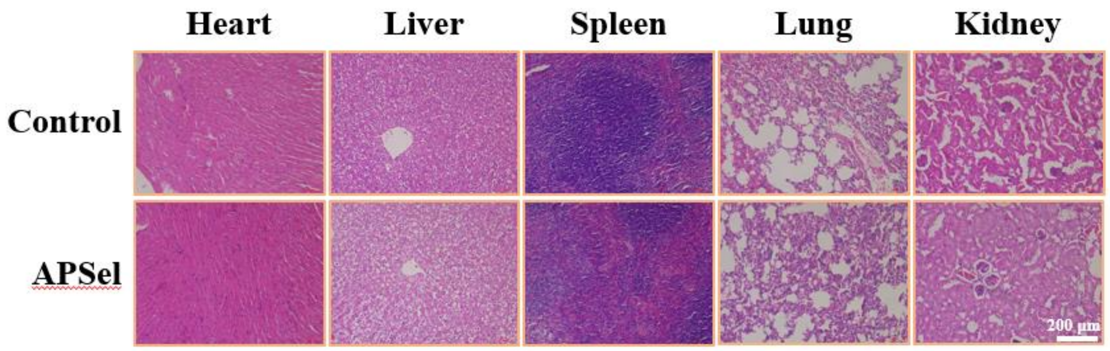


**Fig. S20**. The images of sections from major mouse organs (heart, liver, spleen, lung, kidney) stained by H&E at 24 h after tail vein injection of normal saline (control) or APSel (0.2 mg/kg).

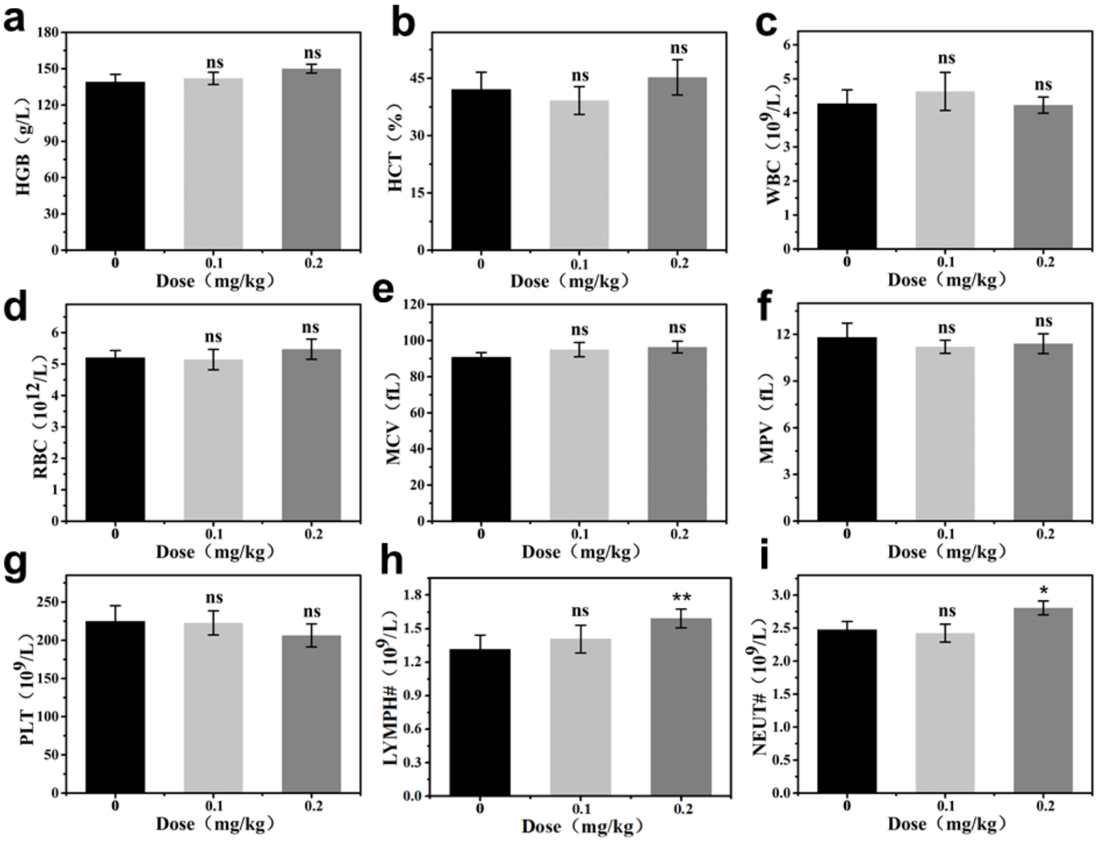


**Fig. S21**. The analysis data of mice blood after tail vein injection of different concentrations of APSel (0, 0.1, 0.2 mg/kg) for 7 days. (a) Hemoglobin (HGB) concentrations; (b) hematocrit (HCT); (c) number of white blood cells (WBC); (d) number of red blood cells (RBC); (e) mean corpuscular volume (MCV); (f) mean platelet volume (MPV); (g) number of blood platelets (PLT); (h) absolute value of lymphocytes ($LYMPH^{\#}$); (i) the absolute value of neutrophils ($NEUT^{\#}$). The error bar represents the standard deviation of three independent measurements. ns: not significant, * $p<0.05$, ** $p<0.01$, as determined by Student's t-test.

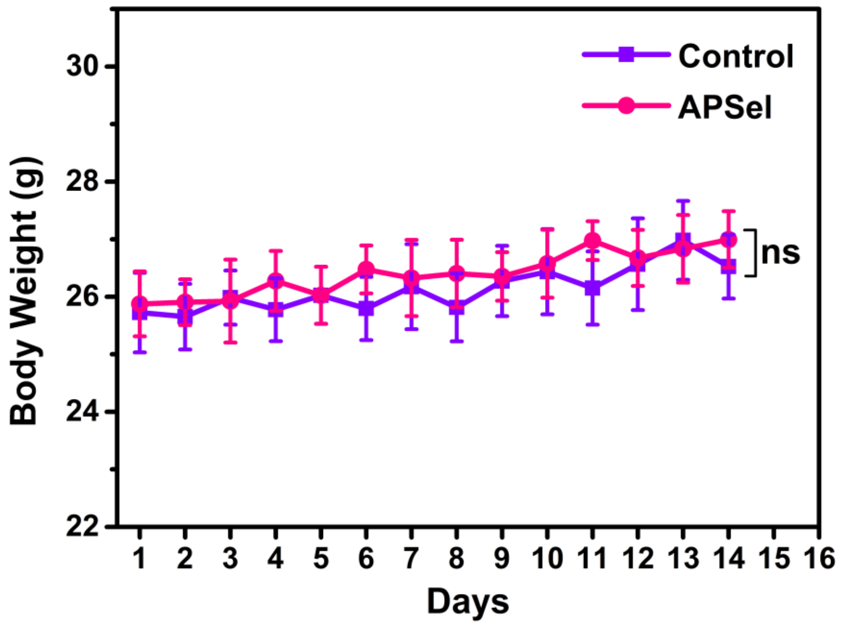


**Fig. S22**. The changes in mice body weight after tail vein injection of normal saline (control) or APSel (0.2 mg/kg) for 14 days. ns: not significant, as determined by Student's t-test.

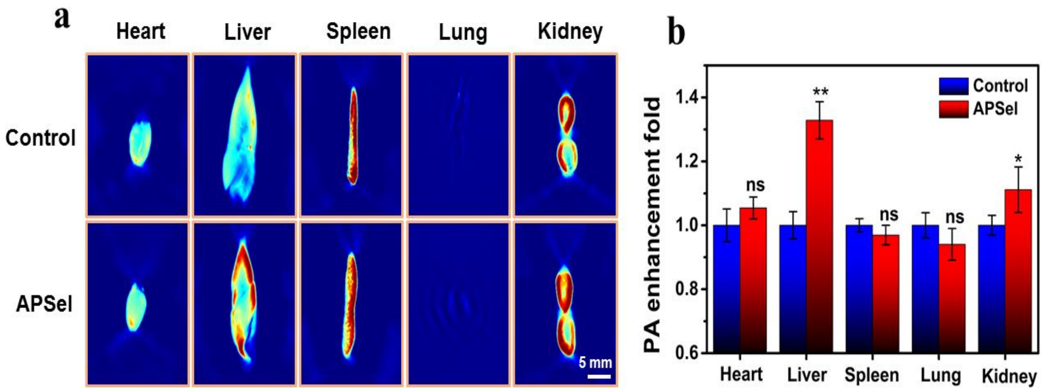


**Fig. S23**. (a) PA imaging of mouse major organs (heart, liver, spleen, lung, kidney) after tail vein injection of normal saline (control group) or APSel (0.1 mg/kg) for 1 h. The excitation wavelength of the PA image is 725 nm. (b) The fold increase of the PA signal for the mouse major organs treated with different conditions (1.0 in control group). The error bar represents the standard deviation of three independent measurements. The statistical analysis was performed using the Student's t-test method, ns: not significant, * $p<0.05$, ** $p<0.01$.

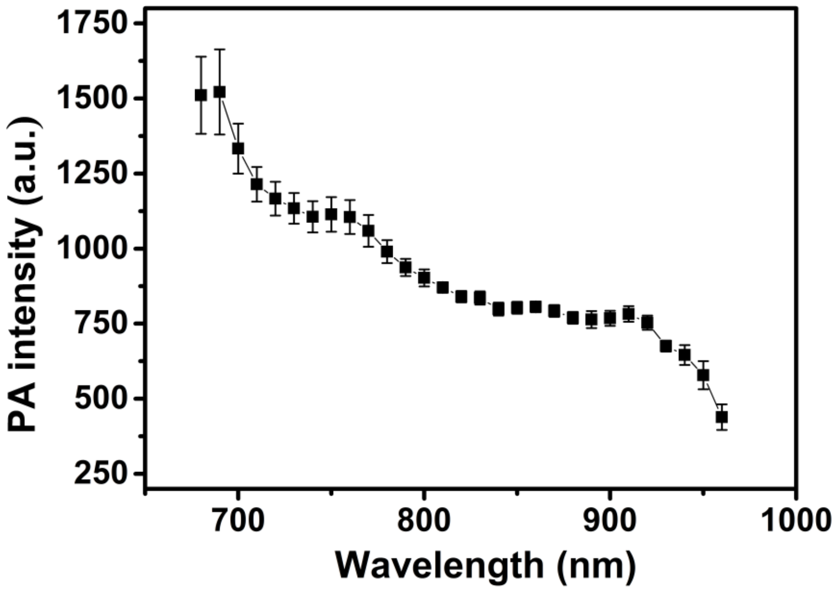


**Fig. S24**. The PA spectrum of isolated mouse liver after injecting normal saline for 1 h. Error bars represent the standard deviation of three independent measurements.

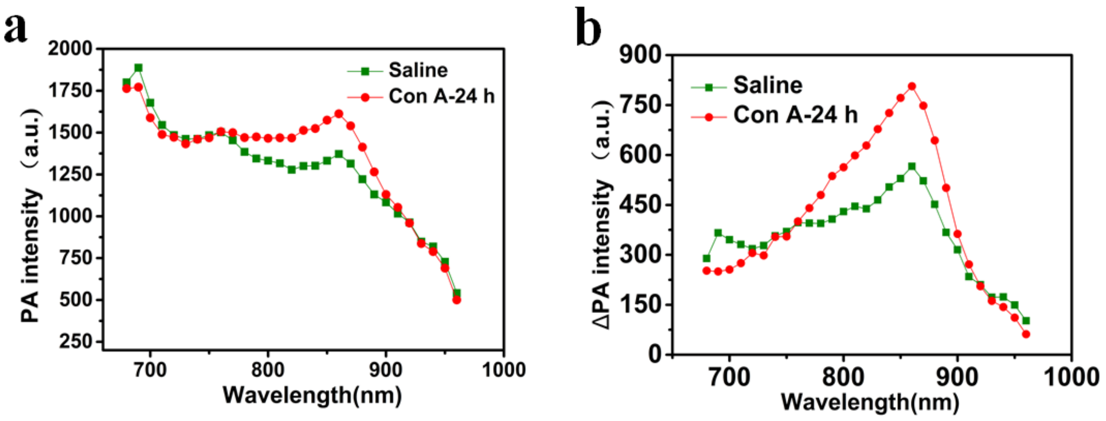


**Fig. S25**. (a) Representative PA spectra of the isolated liver from normal mice and AIH model mice injected with APSel (0.1 mg/kg) at 1 h after injection of Con A (15 mg/kg) 24 h. (b) The PA spectra after removing the background PA signal.

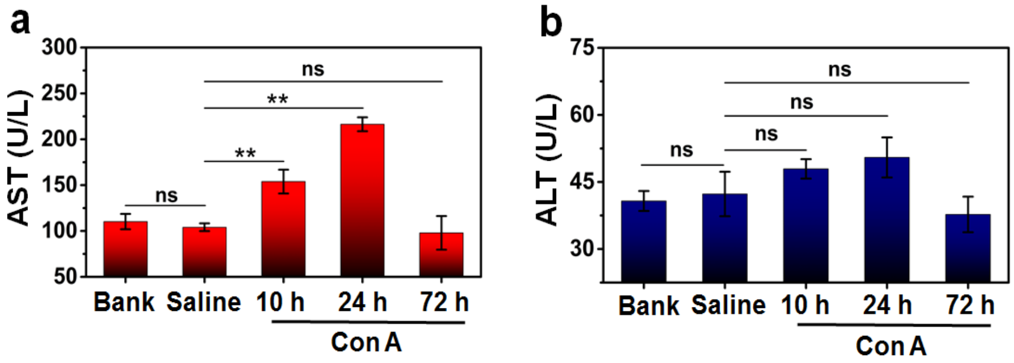


**Fig. S26**. (a) The changes of serum AST. (b) The changes of serum ALT.

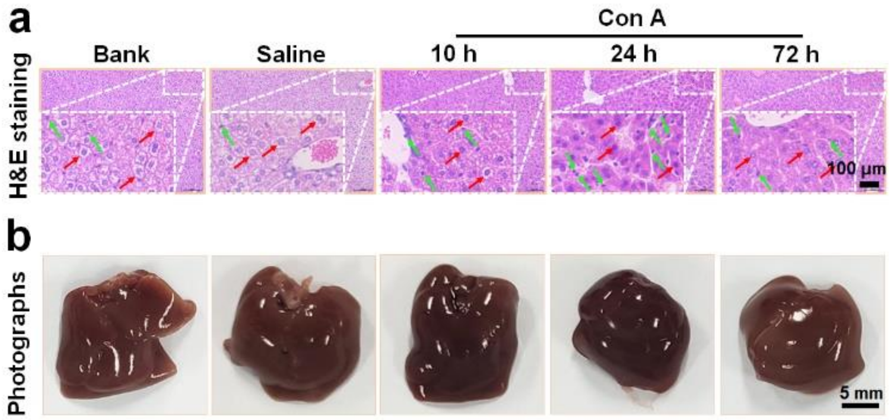


**Fig. S27**. (a) The H&E staining for liver tissues of mice in different conditions, red arrows point to hepatocytes and green arrows point to inflammatory cells. (b) Representative images of entities of mouse liver in different conditions.
